# AIF3 splicing switch triggers neurodegeneration

**DOI:** 10.1186/s13024-021-00442-7

**Published:** 2021-04-14

**Authors:** Shuiqiao Liu, Mi Zhou, Zhi Ruan, Yanan Wang, Calvin Chang, Masayuki Sasaki, Veena Rajaram, Andrew Lemoff, Kalyani Nambiar, Jennifer E. Wang, Kimmo J. Hatanpaa, Weibo Luo, Ted M. Dawson, Valina L. Dawson, Yingfei Wang

**Affiliations:** 1 Department of Pathology, University of Texas Southwestern Medical Center, Dallas, TX 75390 USA; 2 Department of Neurology, University of Texas Southwestern Medical Center, Dallas, TX 75390 USA; 3 Neuroregeneration and Stem Cell Programs, Institute for Cell Engineering, Johns Hopkins University School of Medicine, Baltimore, MD 21205 USA; 4 Departments of Neurology, Johns Hopkins University School of Medicine, Baltimore, MD 21205 USA; 5 Department of Biochemistry, University of Texas Southwestern Medical Center, Dallas, TX 75390 USA; 6 Department of Pharmacology, University of Texas Southwestern Medical Center, Dallas, TX 75390 USA; 7 Solomon H. Snyder Department of Neuroscience, Johns Hopkins University School of Medicine, Baltimore, MD 21205 USA; 8 Physiology, Johns Hopkins University School of Medicine, Baltimore, MD 21205 USA

**Keywords:** AIF, Mitochondrial dysfunction, AIF3 splicing, Neurodegeneration

## Abstract

**Background:**

Apoptosis-inducing factor (AIF), as a mitochondrial flavoprotein, plays a fundamental role in mitochondrial bioenergetics that is critical for cell survival and also mediates caspase-independent cell death once it is released from mitochondria and translocated to the nucleus under ischemic stroke or neurodegenerative diseases. Although alternative splicing regulation of AIF has been implicated, it remains unknown which AIF splicing isoform will be induced under pathological conditions and how it impacts mitochondrial functions and neurodegeneration in adult brain.

**Methods:**

AIF splicing induction in brain was determined by multiple approaches including 5′ RACE, Sanger sequencing, splicing-specific PCR assay and bottom-up proteomic analysis. The role of AIF splicing in mitochondria and neurodegeneration was determined by its biochemical properties, cell death analysis, morphological and functional alterations and animal behavior. Three animal models, including loss-of-function harlequin model, gain-of-function AIF3 knockin model and conditional inducible AIF splicing model established using either Cre-loxp recombination or CRISPR/Cas9 techniques, were applied to explore underlying mechanisms of AIF splicing-induced neurodegeneration.

**Results:**

We identified a nature splicing AIF isoform lacking exons 2 and 3 named as AIF3. AIF3 was undetectable under physiological conditions but its expression was increased in mouse and human postmortem brain after stroke. AIF3 splicing in mouse brain caused enlarged ventricles and severe neurodegeneration in the forebrain regions. These AIF3 splicing mice died 2–4 months after birth. AIF3 splicing-triggered neurodegeneration involves both mitochondrial dysfunction and AIF3 nuclear translocation. We showed that AIF3 inhibited NADH oxidase activity, ATP production, oxygen consumption, and mitochondrial biogenesis. In addition, expression of AIF3 significantly increased chromatin condensation and nuclear shrinkage leading to neuronal cell death. However, loss-of-AIF alone in harlequin or gain-of-AIF3 alone in AIF3 knockin mice did not cause robust neurodegeneration as that observed in AIF3 splicing mice.

**Conclusions:**

We identified AIF3 as a disease-inducible isoform and established AIF3 splicing mouse model. The molecular mechanism underlying AIF3 splicing-induced neurodegeneration involves mitochondrial dysfunction and AIF3 nuclear translocation resulting from the synergistic effect of loss-of-AIF and gain-of-AIF3. Our study provides a valuable tool to understand the role of AIF3 splicing in brain and a potential therapeutic target to prevent/delay the progress of neurodegenerative diseases.

**Supplementary Information:**

The online version contains supplementary material available at 10.1186/s13024-021-00442-7.

## Background

Apoptosis-inducing factor (AIF) is an X-chromosome linked mitochondrial flavoprotein ubiquitously expressed in different tissues and organs [1]. AIF plays a fundamental role in regulating mitochondrial bioenergetics via its N-terminal oxidoreductase domain and also mediates caspase-independent cell death through its C-terminal domain [1–7]. AIF is essential for life and death [8–10]. Emerging evidence showed that at least 23 mutations of AIF have been identified in human patients who commonly developed mitochondrial and neurological deficits [8, 11–21]. Studying functions of AIF and its variants in brain will ameliorate the current understanding of AIF-dependent mitochondrial and neurological diseases.

AIF is initially synthesized as a 67 kDa-precursor in the cytoplasm and imported into mitochondria where it is processed to a 62 kDa-mature AIF protein by removal of 53 amino acids at the N-terminus [22]. AIF mainly locates in the inter membrane space of mitochondria with its N-terminus inserted into the inner membrane [22]. A brain-specific AIF2 isoform, which contains an alternative exon 2B encoding a more hydrophobic mitochondrial inner membrane sorting signal than exon 2A in AIF mRNA, has been identified [23]. AIF2 is assumed to more strongly anchor to the inner membrane than AIF. Three other short AIF isoforms, including AIFsh, AIFsh2 and AIFsh3, have also been reported [2, 3, 6], although their biological functions and the molecular mechanisms accounting for the mRNA splicing remain unknown.

As constitutive deletion of *Aifm1* in mice is embryonic lethal, several conditional AIF knockout mouse models have been developed using tissue-specific Cre recombinases to explore AIF function in vivo. Telencephalon-specific AIF knockout mice have defective cortical development and die by embryonic day 17 (E17), suggesting that AIF is required for neuronal cell survival during the cortical development [24]. Engrailed-Cre-mediated *Aifm1* gene depletion in the midbrain and cerebellum at an early embryonic stage revealed that AIF is critical for both neuron survival and cerebellar development [25]. Harlequin (Hq) mice, which have an 80% reduction in AIF expression due to the proviral insertion into intron 1, develop oxidative stress-mediated neurodegeneration in cerebellum and retina during aging [26]. Apart from its cytoprotective role, AIF also plays an important role in parthanatos (poly(ADP-ribose) polymerase 1 (PARP-1)-dependent cell death) after its release from mitochondria and translocation to the nucleus under ischemic brain injury and neurodegenerative diseases like Parkinson’s disease and Alzheimer’s disease [6, 27–29].

In the present study, we generated a floxed AIF mouse model by targeting exon 3 of *Aifm1* gene with the original goal to understand AIF functions in adult mouse brain. Unexpectedly, the deletion of exon 3 in brain activates AIF splicing mechanism to produce a novel AIF isoform named as AIF3. Importantly, AIF3 is a natural isoform repressed under the physiological conditions but induced in mice and human brain following stroke. AIF3 splicing switch causes neurodegeneration in vitro and in vivo. The molecular mechanism underlying AIF3 splicing-triggered neurodegeneration involves AIF3 splicing-induced mitochondrial dysfunction and AIF3 nuclear translocation, which is attributed to the synergistic effect of both loss-of-AIF and gain-of-AIF3. These findings may provide new mechanistic insights into post-stroke neurodegeneration and other mitochondria-related neurological diseases.

## Methods

### Generation of tissue-specific *Aif* conditional knockout mice

The targeting vector for generating the AIF floxed (fl) allele consists of an 11 kb SphI fragment of the *Aifm1* genomic DNA including 7.8 kb upstream and 3.2 kb downstream of the *Aifm1* exon 3, and two LoxP sites at 114 bp upstream and 190 bp downstream of the *Aifm1* exon 3, respectively. The G418 resistance gene (NEO) flanked by frt sites and the herpes simplex thymidine kinase (TK) gene inserted at 5′ of the targeting construct were used for positive and negative selection, respectively. The targeting construct was electroporated into 129/SV embryonic stem (ES) cells. Homologous recombination introduced a floxed exon 3 along with the Neo/frt cassette in a correctly recombined ES clone. FlpE recombinase was overexpressed in the selected targeted 129/SV ES cells to remove the neomycin cassette, and Southern analysis was performed to screen for clones with the Neo cassette deletion. The selected clones were expanded and injected into C57BL/6 blastocysts to yield chimeric mice. Homozygous AIF^fl/fl^ female mice and hemizygous AIF^fl/Y^ male were backcrossed > 8 times onto the C57BL/6 background prior to initiation of the current studies. For neuron-specific deletion of *Aifm1* exon 3, we crossed AIF^fl/fl^ female with male mice expressing iCre recombinase under the control of the CamKIIα promoter (CamKIIα-iCre) [30, 31] and hemizygous AIF^fl/Y^ male mice were mainly used in this study. Alternatively, we crossed female (AIF^fl/+^, CamKIIα-iCre+) mice with male (AIF^fl/Y^, CamKIIα-iCre-) mice to get homozygous female (AIF^fl/fl^, CamKIIα-iCre+) and its littermate (AIF^fl/fl^, CamKIIα-iCre-) for parallel studies. The body weight and growth curves were investigated in both homozygous AIF^fl/fl^ female mice and hemizygous AIF^fl/Y^ male mice. AIF protein expression was determined by AIF antibodies targeting different antigens (Table 1). Mouse genotyping primers and sizes of polymerase chain reaction (PCR) products were shown in Table 2.

**Table 1:** Antigens of different AIF antibodies

AIF antibody	Antigen	Source
JH532-AIF	aa 598-612 (CEDLNEVAKLFNIHED)	Johns Hopkins-JH532 [32]
Epi-AIF	C-terminus of human AIF	Epitomics (1020-1) [32]
N19-AIF	19 aa at N terminal of AIF exon 4	Santa Cruz (SC-9417)
E1-AIF	aa 1-300 of Human AIF (NP_004199)	Santa Cruz (SC-13116) [32]

**Table 2:** Primers used for genotyping and quantitative PCR

PCR ID	Primers	Sequence	Size (bp)
iCre	CamkIIα-iCre Fw	5’-GACAGGCAGGCCTTCTCTGAA-3’	536
	CamkIIα-iCre Re	5’-CTTCTCCACACCAGCTGTGGA-3’	
	AIFflox Fw1	5’-GCTGGACGTAAACTCCTCTTCAGACCT-3’	Homo: 276
AIF3geno	AIFflox Fw2	5’-CACTGAAAAAGTAAACTTGGCCATAGTGCT-3’	Het: 276, 447
	AIFflox Re	5’-CCTCTCTGGTCTAAAATGCCAAGCAA-3’	WT: 447
mAIF	mAIF-Fw34	5’-ACGGTGCGTGGAAGGAAAAG-3’	1920
	mAIF-Re1953	5’-ATCCCGCCAGGGATGGAAAAG-3’	
mAIF3	mAIF-Fw2E1/4	5’-GAACCGGCTTCCAGCTACAGAG-3’	348
	mAIF-Re648	5’-GTCTGAATTGCAGTGTCTTTG-3’	
Cytb	Cytochrome b Fw	5’-TTCTGAGGTGCCACAGTTATTAC-3’	180
	Cytochrome b Re	5’-GAAGAGGAGGTGAACGATTGC-3’	
RPS2	RPS2 Fw	5’-CTGACTCCCGACCTCTGGAAA-3’	93
	RPS2 Re	5’-GAGCCTGGGTCCTCTGAACA-3’	
hAIF	hAIF-Fw128	5’-TCGTGCGTGAGAGGAAAGG-3’	665
	hAIF-Re761	5’-GTTTGAATCGCAGTGTCTTTG-3’	
hAIF3	hAIF-Fw2E1/4	5’-GAACCGGCTCCCAGCTTCAGAA-3’	338
	hAIF-Re761	5’-GTTTGAATCGCAGTGTCTTTG-3’	
	R26-F2	5’-GCCTCCTGGCTTCTGAGGACCG-3’	WT: 200
Rosa26	R26-R2	5’-TCTGTGGGAAGTCTTGTCCCTCC-3’	KI: 126
	SAR-R	5’-CCTGGACTACTGCGCCCTACAGA-3’	
AIF3KI	mAIF3KI-F	5’-GGGATTGTGCTATGGAACGTC-3’	812
	R26-R2	5’-TCTGTGGGAAGTCTTGTCCCTCC-3’	
	Nestin-WT-F	5’-TTGCTAAAGCGCTACATAGGA-3’	WT: 246
Nestin-Cre	Nestin-R	5’-GCCTTATTGTGGAAGGACTG-3’	Cre: 150
	Nestin-Mu-F	5’-CCTTCCTGAAGCAGTAGAGCA-3’	
	mAIFGSP1-1951	5’-GCTTGTGTATTCCACGATTGGGATTCA-3’	
RACE	mAIFGSP2-1787	5’-GGTAGAAGATGACACCTTTGCCGTAGT-3’	
	mAIFGSP3-1009	5’-CCCGCCGATAACTGTAATTGACTTGAC-3’	
	mAIFGSP4-608	5’-GAGGAGGTCGCATGTATGGCAGTTCAG-3’	

### Generation and validation of tissue-specific conditional AIF3 knockin (KI) mice

The conditional Loxp-eGFP/Stop-Loxp-AIF3KI (LSL-AIF3KI) mice were generated with the CRISPR/Cas9 method by inserting the targeting vector sequence between left (1 kb) and right (4 kb) homology arms into Rosa26 locus as reported previously [33]. First, LoxP-eGFP-stop-SV40 polyA signal-LoxP was assembled by overlapping PCR using the following 4 primers, including Age1-LSL-GFP-F(TACCGGTATAACTTCGTATAGCATACATTATACGAAGTTATCCACCATGGTGAGCAAG), GFP-SV40-R (TTAACAACAACAATTTTAC-TACTTGTACAGCTCGTCCAT), SV40F (AATTGTTGTTGTTAACTTGTTTATT) and SV40R (TCTGCAATCATTTACGCGTGCGATCGCAATTCC), and cloned into pR26 CAG AsiSI/MluI vector (Addgene, #74286) between AgeI and MluI. SV40 polyA signal-LoxP and AsiSI/MluI sites were kept as the same sequence originally on the pR26 CAG AsiSI/MluI vector (Addgene, #74286). Then C-terminal Flag tagged mouse AIF3 cDNA was further inserted into the modified pR26 CAG AsiSI/MluI vector between Asis1 and Mlu1 to generate the targeting vector LSL-AIF3KI. Second, C57BL/6 zygotes were microinjected at UTSW Transgenic Technology Center with Cas9 protein (IDT, Cat# 1081058), sgRNA targeting ROSA26 locus produced as described previously [33] and LSL-AIF3KI targeting vector using the standard procedures reported previously [33]. Injected zygotes were transferred into the oviducts of pseudo-pregnant NMRI female mice to produce pups​. LSL-AIF3KI targeting vector integration into the *Rosa26* locus was determined by PCR using primers pCAGGS-5 (ggttcggcttctggcgtgtgacc) and BGH-R (taggaaaggacagtgggagtg) and verified by Sanger sequencing. LSL-AIF3KI mice were then crossed with CamkIIα-iCre or Nestin-Cre mice to induce AIF3 expression in the presence of AIF in brain. Genotyping was performed by AIF3KI primer pairs, Rosa primers and iCre/nestin-Cre primers as shown in Table 2. Both heterozygous and homozygous AIF3KI mice were used for experiments.

### Animals

Harlequin mouse (#000501; B6CBACa *A*^*w-J*^/*A*-*Aifm1*^*Hq*^/J) and its control mouse (#000051, C57BL/6 J-A^w-J^/J) were purchased from Jackson Laboratory. CamkIIα-iCre mouse line (EM:01153) was purchased from EMMA. Ai-14 mouse (007908, Jackson Lab) was provided by Dr. Woo-Ping Ge at UT Southwestern. All mice were housed and cared in accordance with the NIH Health Guide for the Care and Use of Laboratory Animals in a pathogen-free facility with a 12 h light/dark cycle. UT Southwestern Medical School and The Johns Hopkins Medical Institutions are fully accredited by the American Association for the Accreditation of Laboratory Animal Care (AAALAC). All research procedures performed in this study were approved the Medical Institutions Institutional Animal Care and Use Committee (IACUC) in compliance with the Animal Welfare Act Regulations and Public Health Service (PHS) policy.

### Middle cerebral artery occlusion (MCAO)

Cerebral ischemia was induced by 2 h of reversible MCAO as previously described [27]. Adult male C57BL/6 mice (2 to 4 month-old, 20–28 g) were first injected with Buprenorphine SR (0.5 mg/kg) right before the surgery and then anesthetized with isoflurane during the surgery and body temperature was maintained at 36.5 ± 0.5^o^ C by a feedback-controlled heating system. A midline ventral neck incision was made, and unilateral MCAO was performed by inserting a 7.0 nylon monofilament into the right internal carotid artery 6–8 mm from the internal carotid/pterygopalatine artery bifurcation via an external carotid artery stump. Laser-Doppler was used to measure the occlusion over the lateral parietal cortex in the core of the ischemic regions in the surgery mice. About 80–90% reduction of the blood circulation was controlled and monitored during the surgery. Sham-operated animals were subjected to the same surgical procedure, but the suture was not advanced into the internal carotid artery. Immediately following the surgery, mice were closely monitored in the presence of Buprenorphine SR, which provides up to 48 h of analgesia. Total 21 mice were used for this MCAO study and 18 mice were subjected to either sham or MCAO surgery. All mice waked up within 30 min after the surgery. After 6 h to 3 days of reperfusion, mice were perfused with PBS and stained with triphenyl tetrazolium chloride (TTC) or the brain tissues were harvested directly for RNA and protein analysis.

### Human postmortem brain tissues

The frontal lobes from human postmortem brain without or with diffuse hypoxic ischemic injury or hemorrhage were collected during routine autopsy between 12 and 72 h after death and frozen at -80 °C. The samples were provided by Children’s Medical Center Pediatric Biospecimen Repository as well as Neuropathology tissue bank at UT Southwestern Medical Center. The consent for all autopsy samples includes use of tissue for research purposes. Only deidentified samples were provided with essential information including sex, age and the diagnosis as shown in Supplementary Figure 2e.

### Open field tests

The open field tests were performed to evaluate the locomotor activity and anxiety in a rectangular plastic apparatus (25 cm × 25 cm × 25 cm) using the video-tracking software system (SMART 3.0) from Pan Lab. The field was divided into peripheral zone (5 cm from the edge of walls) and central zone (36% of the total surface of the arena). The time spent in and entries into the center were measured as an anxiolytic indicator [34]. Mice were first transported to the behavior room and left undisturbed to get acclimated to the environment for at least 30 min. Then, each single mouse was placed into the open field facing the center and allowed to explore freely for 8 min. After each experiment, the arena was thoroughly wiped with 70% ethanol solution to remove any feces, urine and odor. Finally, animal travel distance, walk speed and time in each zone for the first 5 min were analyzed using the SMART 3 software.

### Mass spectrometry

Protein gel pieces was reduced and alkylated with DTT (20 mM) and iodoacetamide (27.5 mM). A 0.01 μg/μL solution of trypsin in 50 mM triethylammonium bicarbonate (TEAB) was added to completely cover the gel pieces on ice for 1 h, and then 50 μL of 50 mM TEAB was added to the gel pieces for digestion overnight at 37 °C. Following solid-phase extraction cleanup with an Oasis HLB μelution plate (Waters), the resulting peptides were reconstituted in 10 μL of 2% (v/v) acetonitrile (ACN) and 0.1% trifluoroacetic acid in water. 2 μL of this resulting peptide solution were injected onto an Orbitrap Elite mass spectrometer (Thermo Electron) coupled to an Ultimate 3000 RSLC-Nano liquid chromatography systems (Dionex). Samples were injected onto a 75 μm i.d., 15-cm long EasySpray column (Thermo), and eluted with a gradient from 1 to 28% buffer B over 60 min. Buffer A contained 2% (v/v) ACN and 0.1% formic acid in water, and buffer B contained 80% (v/v) ACN, 10% (v/v) trifluoroethanol, and 0.1% formic acid in water. The mass spectrometer operated in positive ion mode with a source voltage of 2.4 kV and an ion transfer tube temperature of 275 °C. MS scans were acquired at 240,000 resolution in the Orbitrap and up to 14 MS/MS spectra were obtained in the Orbitrap for each full spectrum acquired using collisionally-induced dissociation (CID) with a charge state 1 rejected. Dynamic exclusion was set for 15 s after an ion was selected for fragmentation.

Raw MS data files were analyzed using Proteome Discoverer v2.2 (Thermo), with peptide identification performed using Sequest HT searching against the *Mus musculus* database from UniProt. Fragment and precursor tolerances of 10 ppm and 0.6 Da were specified, and three missed cleavages were allowed. Carbamidomethylation of Cys was set as a fixed modification and oxidation of Met as a variable modification. The false-discovery rate (FDR) cutoff was 1% for all proteins and peptides.

### Rapid amplification of cDNA end analysis (RACE)

5′-RACE was performed using the cDNAs prepared from C57BL/6 mice according to the manufacturer’s recommendation (Invitrogen). For the 5′-RACE, the first round of PCR amplification was done using the adaptor primer (5′-CGACTGGAGCACGAGGACACTGA-3′) and *Aifm1* gene specific reverse primer GSP-Re (5′- GCTTGTGTATTCCACGATTGGGATTCA-3′). The nested PCR was done with the nested adaptor primer (5′- GGACACTGACATGGACTGAAGGAGTA-3′) and *Aifm1* gene specific reverse primer GSP-Nested-Re (5′-GAGGAGGTCGCATGTATGGCAGTTCAG-3′). PCR products were then cloned using zero blunt TOPO PCR cloning kit (Invitrogen) and sequenced.

### Protein expression and purification

Full length mouse AIF cDNA (NM_012019) and AIF3 cDNA missing exons 2–3 (ΔE2–3) but still containing mitochondrial localization signal (MLS) in exon 1 were cloned into cFugw vector at AgeI and EcoRI sites with Flag tag at its C-terminus for mammalian expression system. cDNAs encoding mouse 62 kDa-mature AIF (Δ53 aa) and AIF3 (ΔE1–3, removing MLS) proteins were subcloned into a GST-tagged pGex-6P-1 vector (GE HealthCare) at EcoR I and Xho I restriction sites. All AIF constructs were verified by Sanger sequencing. The proteins were expressed and purified from *E.coli* using glutathione sepharose and the GST-tag was subsequently removed by the precision protease but with 8 aa linker remaining on the AIF. To exclude the interference from the tag, non-tagged recombinant AIFs were used for the in vitro biochemical determinations. The protein purity, stability and concentrations were determined in the Coomassie blue gel using bovine serum albumin (BSA, 98% purity) as the standard.

### Lentivirus construction and virus production

Full-length cDNAs of AIF-Flag and AIF3-Flag (Supplementary Figure 1f) were subcloned into a lentiviral cFugw vector at Age I and EcoR I restriction sites, and their expression were driven by human ubiquitin C (hUBC) promoter. The lentivirus was produced by transient transfection of the recombinant cFugw vector into 293 T cells with three packaging vectors: pLP1, pLP2 and pVSV-G (1.3: 1.5:1:1.5). The viral supernatants were collected at 48 h and 72 h after transfection and concentrated by ultracentrifuge for 3 h at 50,000 g.

### Mouse embryonic fibroblast (MEFs) culture

MEFs were prepared from the time pregnant AIF^fl/fl^ female mice carrying embryos at E13 after crossing with AIF^fl/Y^ male mice as described previously and maintained in Dulbecco’s Modified Eagle Medium (DMEM) [35]. Then MEFs were infected with either polyomavirus simian virus 40 (SV40) or SV40 together with Cre lentivirus. Single MEF clones were picked and AIF3 expression was verified by western blot after Cre lentiviral infection.

### Mouse cortical neuron culture, transfection, lentiviral transduction and cytotoxicity

Primary neuronal cultures from the cortex were prepared as described previously [36]. Briefly, the cortex was dissected and the cells dissociated by trituration in modified Eagle’s medium (MEM), 20% horse serum, 30 mM glucose and 2 mM L-glutamine following a 10 min digestion in 0.027% trypsin/saline solution (Gibco BRL). Neurons were plated on 15 mm multiwell plates coated with polyornithine or on coverslips coated with polyornithine. Neurons were maintained in MEM with 10% horse serum, 30 mM glucose, and 2 mM L-glutamine in a 7% CO_2_ humidified 37 °C incubator. The growth medium was refreshed twice per week. In mature cultures, neurons represent 70–90% of the total number of cells. In DIV 7–9, neurons were infected by lentivirus carrying AIF-Flag, AIF3-Flag, Cre-GFP or GFP (1 × 10^9^ T.U./ml) for 72 h. Cell viability was determined at DIV 10–13 by unbiased objective computer-assisted cell counting after staining of all nuclei with 7 μM Hoechst 33342 (Invitrogen) and dead cell nuclei with 2 μM propidium iodide (Invitrogen). The numbers of total and dead cells were counted with the Axiovision 4.3 software (Carl Zeiss). At least three independent experiments using at least six separate wells were performed with a minimum of 15,000–20,000 neurons or cells counted per data point.

### Determination of NADH oxidase activity and FAD binding of AIF protein

The redox activity of AIF protein was determined as described [29]. In brief, the redox activity was measured at room temperature in the substrate solution containing 250 μM NADH in air-saturated 50 mM Tris-HCl, pH 8.0. The decrease in absorbance at OD340 nm was monitored right after the addition of recombinant AIF and AIF3 (3 μM in 50 μl reaction volume) into the substrate solution. FAD (25 nM) binding to AIF was monitored at 1 μM concentration of AIF and AIF3 by wave length scanning ranging from 250 to 800 nm using a Beckman Coulter DU800 UV/Vis spectrophotometer (Fullerton).

### DNA gel retardation assay

AIF binding-mediated DNA mobility retardation was performed as described [37]. Recombinant AIF, AIF3 and bovine serum albumin (BSA,10 μg) were incubated with 200 ng 1 kb plus DNA ladder (Fermentas) for 30 min at room temperature and loaded on 2.5% agarose gel prestained with ethidium bromide (1 μg /mL). Mobility shift was visualized under the Alpha Innotech UV illuminator (San Leandro).

### Immunoblotting

The proteins were separated on denaturing polyacrylamide gel electrophoresis (SDS-PAGE) and transferred to nitrocellulose membrane. The membrane was blocked and incubated overnight with primary antibodies: mouse anti-Flag (Sigma, F3165), rabbit anti-AIF(JHU532), anti-Epi-AIF (Epitomics, 1020–1), anti-E1-AIF (Santa Cruz, SC-1316), anti-N19-AIF (Santa Cruz, SC-9417), or actin (Proteintech, 66009-1-AP) at 4 °C, followed by donkey anti-mouse or goat anti-rabbit IgG conjugated to HRP for 1 h at room temperature. After washing, the immune complexes were detected by the SuperSignal West Pico Chemiluminescent Substrate (Pierce).

### Immunocytochemistry, immunohistochemistry and confocal microscopy

For immunocytochemistry, cells were fixed with 4% paraformaldehyde, and permeabilized with 0.05% Triton X-100 and blocked with 3% BSA in phosphate buffered saline (PBS). AIF was visualized by 2 μg/mL mouse anti-Flag/Cy2 affinipure donkey anti-mouse IgG (Jackson-immunoResearch) or rabbit anti-AIF (JHU532 or Santa Cruz SC-1316)/Cy3 affinipure donkey anti-rabbit IgG (Jackson-immunoResearch). For immunohistochemistry, brain sections were fixed with 4% paraformaldehyde, permeabilized with 0.1% Triton X-100 and blocked with 10% normal donkey serum in PBS. Brain sections were then incubated with the antibodies against AIF (JHU532 or Santa Cruz SC-1316), Calbindin (Sigma, C9848), CUX1 (Proteintech, 11733-1-AP), TBR1 (Proteintech, 20932-1-AP) and anti-NeuN (Sigma, ABN90) followed by Cy2 affinipure donkey anti-mouse IgG or Cy3 affinipure donkey anti-rabbit IgG. Immunofluorescent and immunohistochemical analysis were carried out by using a LSM510 confocal laser scanning microscope (Carl Zeiss) as described previously [29].

### Electron microscopy

Mice were first perfused through the heart with PBS (pH 7.4) for 30 s, and then perfused with 3% paraformaldehyde, 1.5% glutaraldehyde, 100 mM cacodylate and 2.5% sucrose (pH 7.4), followed by postfixing for 1 h. Mouse cortex, hippocampus and cerebellum sections were processed as described [38]. They were postfixed in Palade’s OsO_4_, en bloc stained in Kellenberger’s uranyl acetate, dehydrated, and flat-embedded in epon. 80 nm en face sections were prepared on a Leica UCT ultramicrotome, collected onto 400 mesh high transmission nickel grids, and post stained with lead and uranyl acetate. Images were taken by a Philips EM 410 TEM equipped with a Soft Imaging System Megaview III digital camera.

### Mitochondrial DNA copy measurement

Total DNA was isolated from different tissues. The amount of mitochondrial DNA relative to nuclear genomic DNA was determined by quantitative real-time PCR using primers (Table 2) for mitochondrial DNA-encoded cytochrome b and nuclear DNA-encoded ribosomal protein S2 (RPS2). Relative mitochondrial DNA levels were calculated based on the threshold cycle (Ct) as 2^-Δ(ΔCt)^, where ΔCt = Ct_Cytochrome b_ – Ct_RPS2_ and Δ(ΔCt) = ΔCt_AIFΔEx3_ – ΔCt_WT_.

### Intracellular adenosine triphosphate (ATP)

ATP levels were measured using an ATP assay kit (Promega) according to the manufacturer’s instructions. Luminescence was measured using a GloMAX 20/20 single tube luminometer (Promega) and normalized to the protein concentration.

### Measurement of oxygen consumption rate (OCR)

The OCR was measured using a cartridge containing an optical fluorescent O_2_ sensor in a Seahorse Bioscience XF24 Extracellular Flux Analyzer (Seahorse Bioscience). Mouse cortical neurons prepared from Hq mice or AIF^fl/Y^/AIF^fl/fl^ mice were seeded and cultured in XF24-well microplates at 2.5 × 10^5^ cells per well for 3 days and infected with lentivirus as indicated for 3 days. OCR was measured after equilibration in assay medium containing 30 mM glucose for 1 h. Baseline rates were measured three times. Three testing chemicals, including oligomycin, FCCP, and rotenone, were preloaded in the reagent delivery chambers of the sensor cartridge and then pneumatically injected into the wells to reach the desired final working concentrations. After 2 min of mixing, postexposure OCR was measured for three times. The averages of three baseline rates and three test rates were used for data analysis. OCR was shown in the unit of picomoles per minute.

### Nissl staining

Nissl staining was performed using standard methods as previously described [39]. For immunohistochemical analysis, mice were anesthetized with a lethal dose of pentobarbital (100 mg/kg) and perfused with 4% paraformaldehyde (Sigma). Brains were extracted, postfixed and cryoprotected in 30% sucrose. Free-floating sections (30 μm) were harvested with a microtome and processed for immunohistochemical analysis.

### Experimental design and statistical analysis

AIF3 splicing mice and AIF3 conditional KI mice were housed with their own littermate controls in the same cage and identified by the unique number ID with genotyping. Animal experiments were performed in a blind fashion and decoded when data analysis. MCAO experiments with C57BL/6 mice were performed in a randomized fashion. All experiments were repeated at least three times. All statistical evaluation was carried out using GraphPad Prism 8 software. Student’s *t*-test was performed between two groups in Figs. 2l, 3e-h, 5c, 8f and 10f-g. One-way analysis of variance (ANOVA) was performed in Figs. 2i, 4c, 6b, 8c, and 9c, e-f and two-way ANOVA were performed in Figs. 3a-d, 8h and 9a-b by GraphPrism software within multiple groups. Data are shown as mean ± S.E.M., *p* < 0.05 is considered significant.

## Results

### Identification of a novel AIF isoform-AIF3

**Fig. 1:**
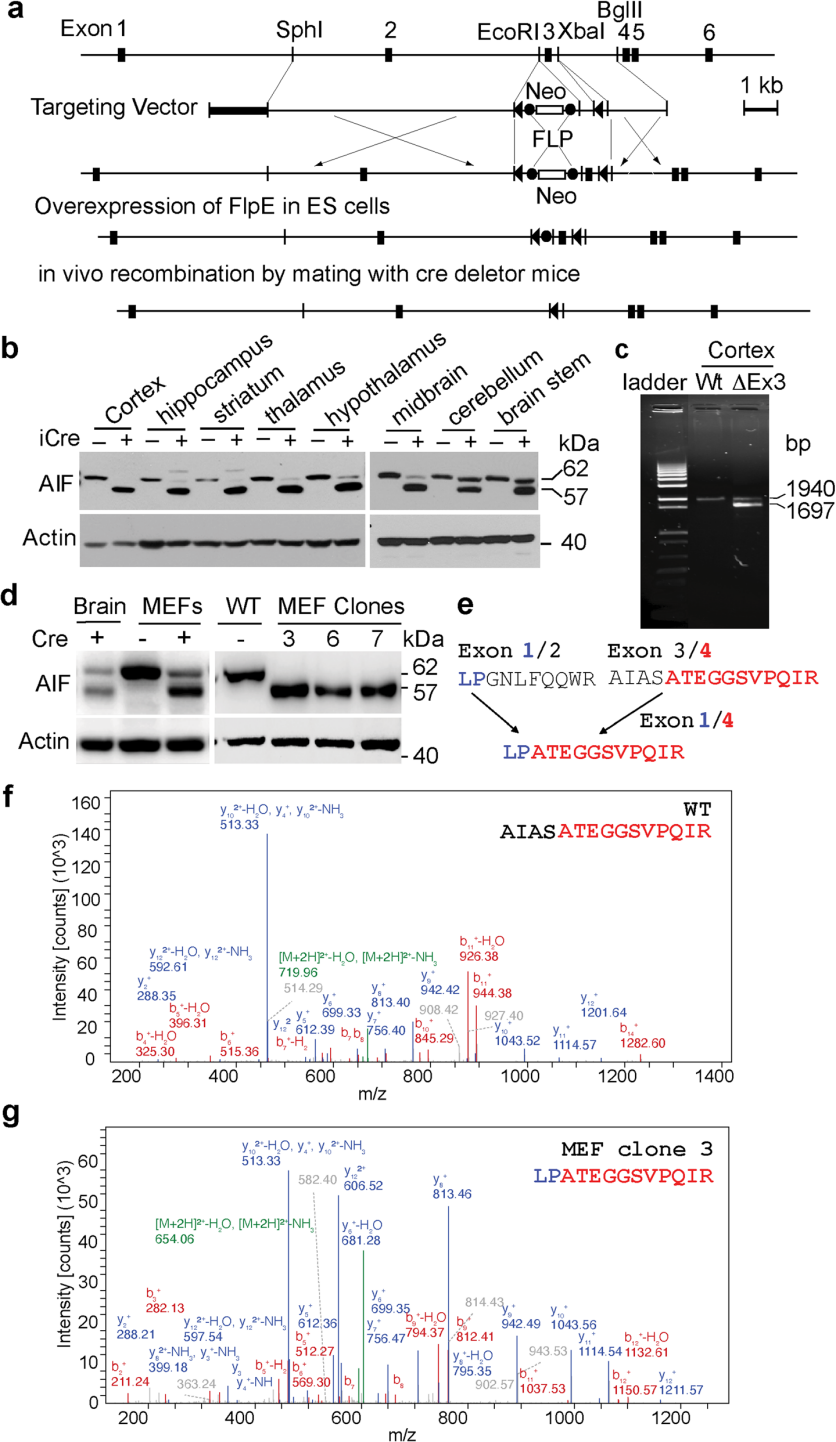
Identification of a novel AIF isoform-AIF3. **a** Strategy for conditional deletion of exon3 of mouse *Aifm1* gene. A partial restriction map of *Aifm1* gene locus and the targeting vector are shown on the first and second lines. Exons of the murine *Aifm1* gene are indicated as a filled box and labeled. Exon 3 is flanked by *lox*P sequences which are indicated by triangles. The G418 resistance gene (Neo) flanked by *frt* sites is indicated with the open box. The PGK-herpes simplex thymidine kinase (TK) indicated by thick line is inserted at the 5′ end of the targeting vector. Restriction endonuclease sites used for cloning are shown (SphI, EcoRl; XbaI; BglII). Homologous recombination introduces a floxed exon 3 along with the Neo cassette, which is excised by transient overexpression of flpE recombinase in a correctly recombined ES clone. The floxed exon 3 in the *Aifm1* allele is further excised by mating with mice expressing Cre recombinase in a brain specific manner. **b** Expression of AIF3 in different brain regions in AIF^fl/Y^ mice after CamKIIα-iCre recombination at P70 determined by JHU-532 AIF antibody. The recombinant 62 kDa mature AIF (Δ53 aa) and 57 kDa truncated AIF (Δ103 aa), which have an extra 8 aa linker, were used as markers. **c** mRNA expression and sequence analysis of AIF3 using AIF gene specific primers on its exon 1 and exon 16 (PCR ID: mAIF, Table 2) and cDNA prepared from WT (AIF^fl/Y^/CamKIIα-iCre-) and AIF3 (AIF^fl/Y^/CamKIIα-iCre+) mice at P20 as the template. **d** Establishment of AIF3 MEFs single cell lines from AIF^fl/Y^/AIF^fl/fl^ mice determined by E1-AIF antibody. **e**, AIF peptides bridging Exons 1/2, Exons 3/4, and Exons 1/4. **f** & **g** MS/MS spectra for AIF and AIF3 peptides bridging Exons 3/4 and Exons 1/4, respectively

To explore AIF functions in adult brain, exon 3 of murine *Aifm1* was flanked by *lox*P (AIF^fl/fl^) (Fig. 1a and Supplementary Figure 1a), and AIF^fl/fl^ mice were crossed with *CamKIIα-*iCre mice [30]. The 62 kDa mature AIF protein in AIF^fl/Y^/CamKIIα-iCre+ mice was significantly reduced in the cortex, hippocampus, striatum, thalamus, hypothalamus, and midbrain, but not in the cerebellum and brain stem (Fig. 1b). This is consistent with the expression distribution of the *CamKIIα* gene predominantly in the adult forebrain [31]. Unexpectedly, an additional AIF protein of 57 kDa was detected in all brain regions of AIF^fl/Y^/CamKIIα-iCre+ mice but not in AIF^fl/Y^/CamKIIα-iCre- littermates, which was first observed at postnatal day 20 (P20) (Supplementary Figure 1b) and persistent for about 3 months till their death (Fig. 1b). The 57 kDa protein was recognized by at least three different AIF antibodies targeting different amino acid sequences of AIF (Fig. 1b, Supplementary Figure 1b, c, and Table 1). However, it was not observed in other organs of AIF^fl/Y^/CamKIIα-iCre+ mice, including heart, lung, liver and limb (Supplementary Figure 1d), or in any brain regions of CamKIIα-iCre+ control mice (Supplementary Figure 1e). These results suggest that the 57 kDa protein may be a novel AIF isoform.

To ascertain whether a novel AIF isoform was induced in AIF^fl/Y^/CamKIIα-iCre+ mice, we prepared cDNAs from AIF^fl/Y^/CamKIIα-iCre+ mice brain and their littermate controls (AIF^fl/Y^/CamKIIα-iCre-), and performed PCR using primers specific to exon 1 and 16 of the *Aifm1* gene (PCR ID-mAIF, Table 2). A second smaller PCR product of 1697 bp was detected in the cortex of AIF^fl/Y^/CamKIIα-iCre+ mice in addition to the full-length AIF cDNA (1940 bp) (Fig. 1c). Sanger sequencing analysis revealed that this smaller cDNA encoded AIF lacking exons 2 and 3 (Fig. 1c and Supplementary Figure 1f, g), and is referred to as AIF3 to distinguish it from the other two previously characterized AIF isoforms.

To further determine whether this 57 kDa AIF3 protein was a novel AIF isoform but not a degradation product, we prepared MEFs from AIF^fl/Y^/AIF^fl/fl^ mouse embryos at E13, and infected MEFs with Cre lentivirus to induce endogenous AIF3 splicing and established multiple immortalized single clones expressing AIF3 splicing instead of AIF (Fig. 1d). Then we immunoprecipitated AIF proteins from AIF^fl/Y^/Cre-;AIF^fl/fl^/Cre- MEFs (wild-type, WT) and AIF^fl/Y^/Cre+;AIF^fl/fl^/Cre + clone 3 MEFs for bottom-up proteomic analysis. AIF peptides, especially the peptide linkages between exon 4 and its upstream exons including exons 1 and 3, were analyzed (Fig. 1e). The peptide link LPATEGGSVPQIR between exons 1 and 4 was uniquely detected in AIF^fl/Y^/Cre+;AIF^fl/fl^/Cre + clone 3 MEFs, but not in WT MEFs (Fig. 1f, g). Conversely, the peptide link AIASATEGGSVPQIR between exons 3 and 4 was only detected in WT MEFs (Fig. 1f, g). Peptides encoded by *Aifm1* exons 4–16 were observed in both WT and AIF^fl/Y^/Cre+;AIF^fl/fl^/Cre + clone 3 MEFs. Collectively, mRNA analysis and bottom-up proteomic analysis revealed the presence of a novel 57 kDa AIF3 isoform in the brain of AIF^fl/Y^/CamKIIα-iCre+ and AIF^fl/fl^/CamKIIα-iCre+ mice.

### AIF3 is a natural splicing isoform and induced following stroke in mouse and human

**Fig. 2:**
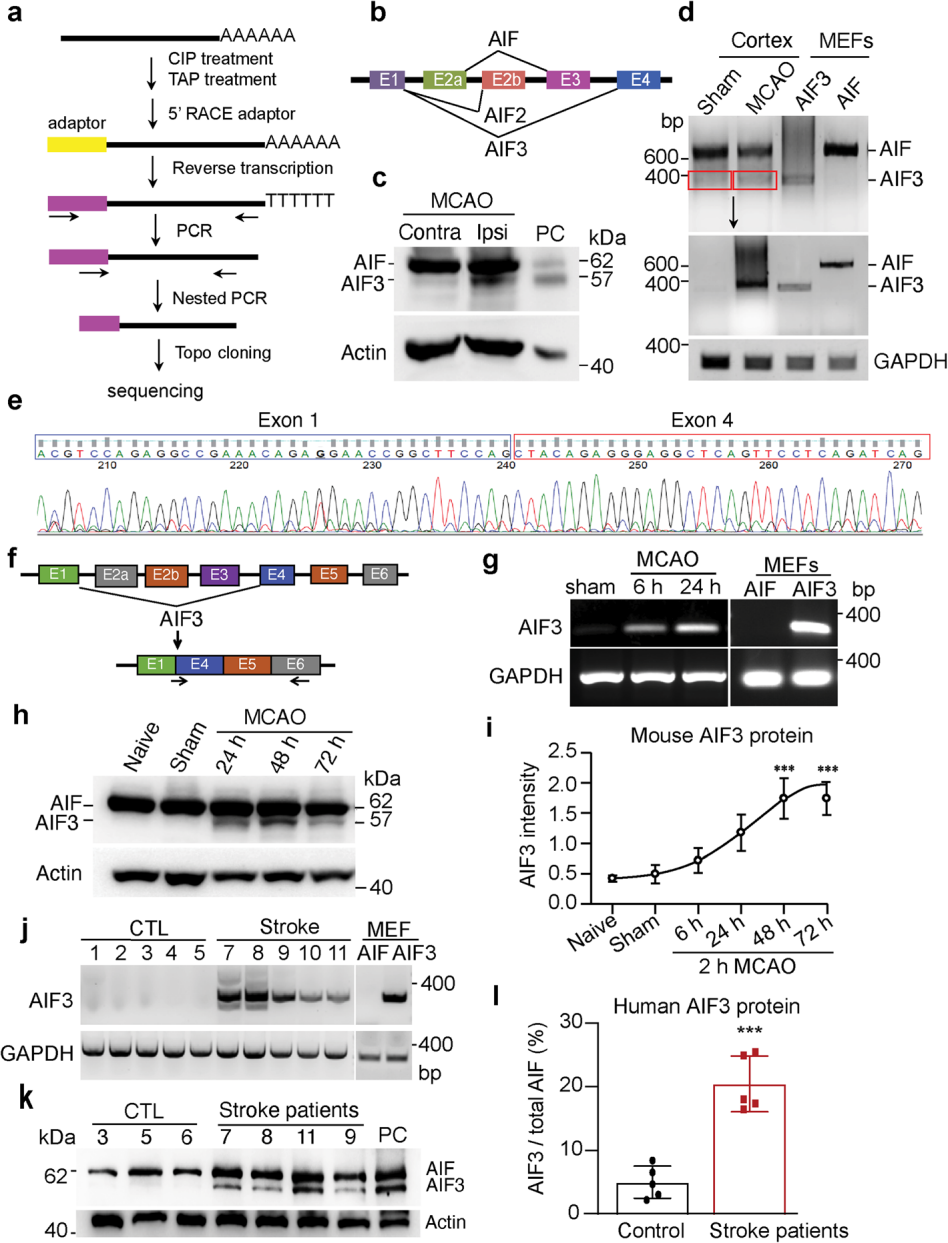
AIF3 was induced following ischemic stroke in mouse model and human patients. **a** The 5′-RACE scheme of AIF cDNAs from C57BL/6 mouse brain. **b** All three AIF isoforms identified by 5′ RACE. **c** Expression of AIF3 protein in C57BL/6 mouse brain 24 h after 2 h MCAO. Contra, contralateral; Ipsi, ipsilateral. PC (positive control), cerebellum lysate of AIF3 (AIF^fl/Y^/CamKIIα-iCre+) mice. **d** Expression of AIF3 mRNA in ischemic stroke mouse cortex using the common AIF/AIF3 primers (mAIF-Fw34/Fe648, Table 2). Top image is the 1st PCR with 20 cycles, middle image is the 2nd PCR using AIF3 from 1st PCR as template, and bottom image is GAPDH. **e** Sanger sequencing of AIF3 mRNA in ischemic stroke mouse cortex prepared from **d**. **f** Scheme for AIF3 mRNA specific primer design. **g** Expression of AIF3 mRNA in ischemic stroke mouse cortex using AIF3 specific primers (PCR ID: mAIF3, Table 2). MEFs were used as controls. **h** Expression of AIF3 protein in ischemic stroke mouse cortex at 24 h, 48 h and 72 h following 2 h MCAO determined by AIF E1 antibody. **i** Quantification of AIF3 protein expression in ischemic stroke mouse cortex at 6 h–72 h following 2 h MCAO. AIF3 protein levels were normalized to actin and presented as the relative intensity. Data are shown as mean ± S.E.M and analyzed for statistical significance by one-way ANOVA. *n* = 3. ***p < 0.001. **j** Expression of AIF3 mRNA in human cortex with stroke using AIF3 specific primers (PCR ID: hAIF3, Table 2). **k** Expression of AIF3 protein in the cortex of human stroke patients. PC, cerebellum lysate of AIF3 (AIF^fl/Y^/CamKIIα-iCre+) mice. **l** Quantification of AIF3 expression protein in human stroke patients. Data are shown as mean ± S.E.M and analyzed for statistical significance by Student *t* test. *n* = 5. ***p < 0.001

To determine whether AIF3 is a natural splicing isoform, 5′ RACE assay, a method to detect the natural minor isoform, was performed using cDNAs prepared from C57BL/6 mouse brain to amplify the 5′ ends of AIF (Fig. 2a). All three AIF isoforms, including AIF, AIF2 and AIF3, were identified by 5′ RACE (Fig. 2b), indicating that AIF3 is a natural splicing isoform. Although AIF3 mRNA and protein levels were below detection limits in mouse brain under physiological conditions (Fig. 1b, c), we found that AIF3 protein was induced in the ipsilateral brain 24 h following 2 h middle cerebral artery occlusion (MCAO) (Fig. 2c & Supplementary Figure 2a). Using primers recognizing AIF exons 1 and 5 (mAIF-Fw34/Re648, Table 2), AIF mRNA was detected in both stroke and sham groups, but AIF3 mRNA was upregulated in brain with ischemic stroke (Fig. 2d). The resulting AIF3 PCR product from exons 1 to 5 was confirmed by Sanger sequencing. The link sequence between exons 1 and 4 was obviously detected in ischemic mouse brain (Fig. 2e). To avoid the interference from AIF, we designed an AIF3-specific PCR primer (mAIF-Fw2E1/4) recognizing the linkage of exons 1 and 4 (Fig. 2f, g). Using AIF3 specific PCR primers (PCR ID-mAIF3, Table 2), we found that AIF3 mRNA was induced in mouse brain at 6 h reperfusion after 2 h MCAO and further elevated at 24 h reperfusion, whereas no AIF3 mRNA was obviously detected in sham mice (Fig. 2g). Consistent with mRNA induction, AIF3 protein levels were increased 24 h following stroke and its upregulation persisted for at least 72 h in mouse brain (Fig. 2h, i). In contrast, AIF3 was not induced in cultured cortical neurons following treatment with N-methyl-D-aspartate (NMDA, 500 μM for 5 min or 100 μM for 2 h), staurosporine (STS, 1 μM), oxygen-glucose deprivation (OGD, 90 min), or hypoxia (1% O_2_ for 24–72 h) (Supplementary Figure 2b, c, d).

To determine the potential clinical relevance of AIF3, AIF3 expression was analyzed in brain frontal cortex from both male and female patients at ages varying from 20 days to 73 years who suffered either hypoxic-ischemic or hemorrhagic stroke (Supplementary Figure 2e). Using AIF3 specific primers (PCR ID-hAIF3, Table 2), the expression of AIF3 mRNA was observed in all 5 human patients tested, which was further confirmed by Sanger sequencing (Fig. 2j, Supplementary Figure 2e). AIF3 protein expression was also increased in these human patients with hypoxic-ischemic injury (Fig. 2k, l, Supplementary Figure 2e). In contrast, little AIF3 mRNA and protein expression was detected in control human brain tissues (Fig. 2j, k, l). These data indicate that AIF3 is a novel AIF splicing isoform and induced in brain following hypoxic-ischemic injury in both mice and humans.

### AIF3 splicing switch causes progressive neurodegeneration in vivo

**Fig. 3:**
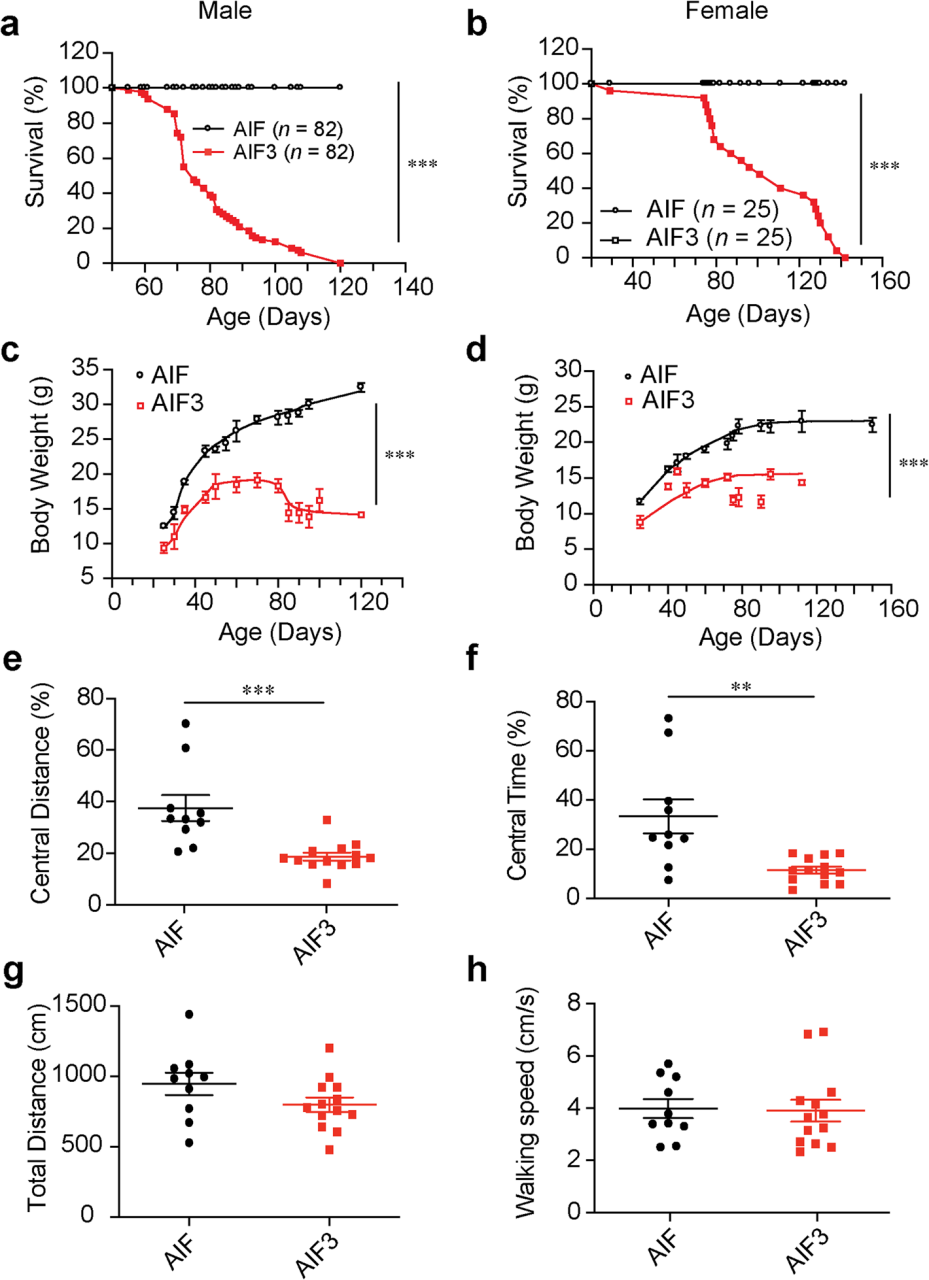
Characterization of the growth and behavior of AIF and AIF3 splicing mice. **a-b** Survival curves of male (a, AIF^fl/Y^/CamKIIα-iCre+, *n* = 82) and female (b, AIF^fl/fl^/CamKIIα-iCre+, *n* = 25) AIF3 splicing mice and their littermate AIF controls (AIF^fl/Y^/CamKIIα-iCre- and AIF^fl/fl^/CamKIIα-iCre-). Data were shown as mean and analyzed for statistical significance by two-way ANOVA. *** p < 0.001. **c-d** Measurement of the body weight of male (c, *n* = 20) and female (d, *n* = 20) AIF3 splicing mice and their littermate AIF controls. Data are shown as mean ± S.E.M. and analyzed for statistical significance by two-way ANOVA. *** p < 0.001. **e-h** Open field tests of AIF3 (*n* = 13) and their littermate controls (*n* = 10) at P60–75. Central zone travel distance (**e**), percentage of time in the Central zone (**f**), total travel distance (**g**) and walk speed (**h**) were analyzed. Data are shown as mean ± S.E.M. and analyzed for statistical significance by Student *t* test. ** p < 0.01; *** p < 0.001, *n* = 10–13

To determine the role of AIF3 splicing in brain, we took advantage of AIF^fl/Y^/CamKIIα-iCre+ mice, in which AIF3 is spliced endogenously instead of AIF in brain. Comparing hemizygous male AIF3 splicing mice (AIF^fl/Y^/CamKIIα-iCre+) and homozygous female AIF3 splicing mice (AIF^fl/fl^/CamKIIα-iCre+) to their littermates (AIF^fl/Y^/CamKIIα-iCre-, or AIF^fl/fl^/CamKIIα-iCre-), we found that both male and female AIF3 splicing mice were born at the expected Mendelian frequency and survived for the first 60 days (Fig. 3a, b, c, d). However, all AIF3 splicing mice died between P60 and P140 (Fig. 3a, b). None of their littermate controls died before P140. Moreover, all AIF3 splicing mice were smaller than their littermate controls and showed severe weight loss at P60-P90 (Fig. 3c, d). Open field tests were performed to evaluate the spontaneous locomotor activity and anxiety-like behaviors. We found that the total travel distance and the speed of mouse walking were not obviously changed at P60-P75. However, the travel paths and percentage of time spent in the center zone were significantly decreased in AIF3 groups compared to their littermates (Fig. 3e, f, g, h), indicating increase of anxiety in AIF3 splicing mice.

**Fig. 4:**
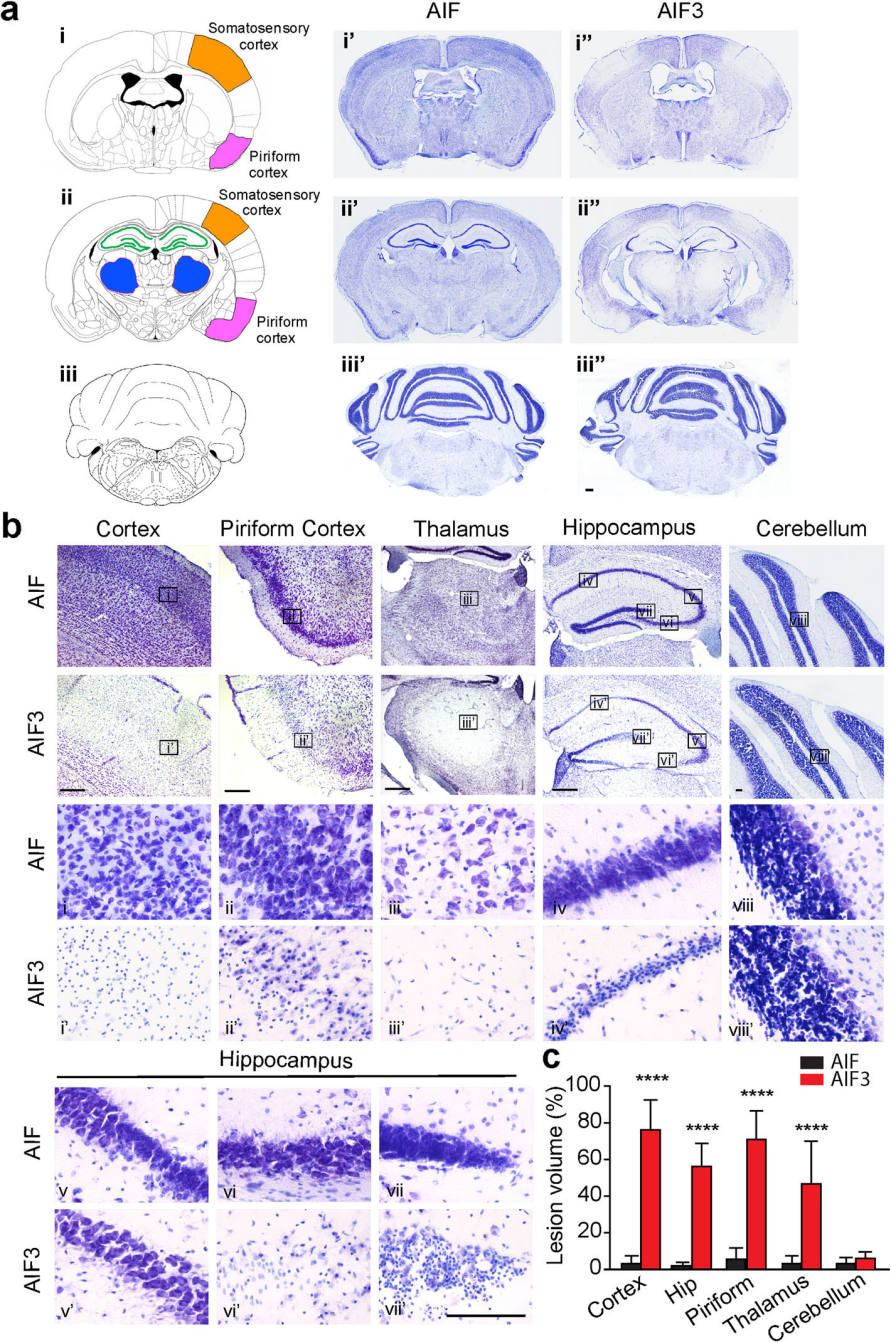
AIF3 splicing caused severe neurodegeneration in the forebrain of 3-month-old mice. **a** Representative overview images of neurodegeneration in mouse brain, including somatosensory cortex, piriform cortex, thalamus and hippocampus (i-ii, i’-ii’, i”-ii”) as well as cerebellum (iii, iii’, iii”). i-iii, drawing images for coronal mouse brain sections. Somatosensory (orange), piriform cortex (pink) and thalamus (blue) are highlighted. i’-iii’, Nissl staining images of the littermate control AIF mouse (AIF^fl/Y^/CamKIIα-iCre-). i”-iii”, Nissl staining images of AIF3 splicing mouse (AIF^fl/Y^/CamKIIα-iCre+). **b** Nissl staining of somatosensory cortex (i, i’), piriform cortex (ii, ii’), thalamus (iii, iii’), hippocampus (iv-vii, iv’-vii’) and cerebellum (viii, viii’) from AIF3 splicing mice and their littermate AIF mice. The boxes in i-viii and i’-viii’ are shown at higher magnification. Scale bar, 200 μm. **c** Quantification of the brain lesion volume (*n* = 5). Cortex, Somatosensory cortex; Hip, hippocampus; Piriform, piriform cortex. Data are shown as mean ± S.E.M. and analyzed for statistical significance by one-way ANOVA. **** p < 0.0001

**Fig. 5:**
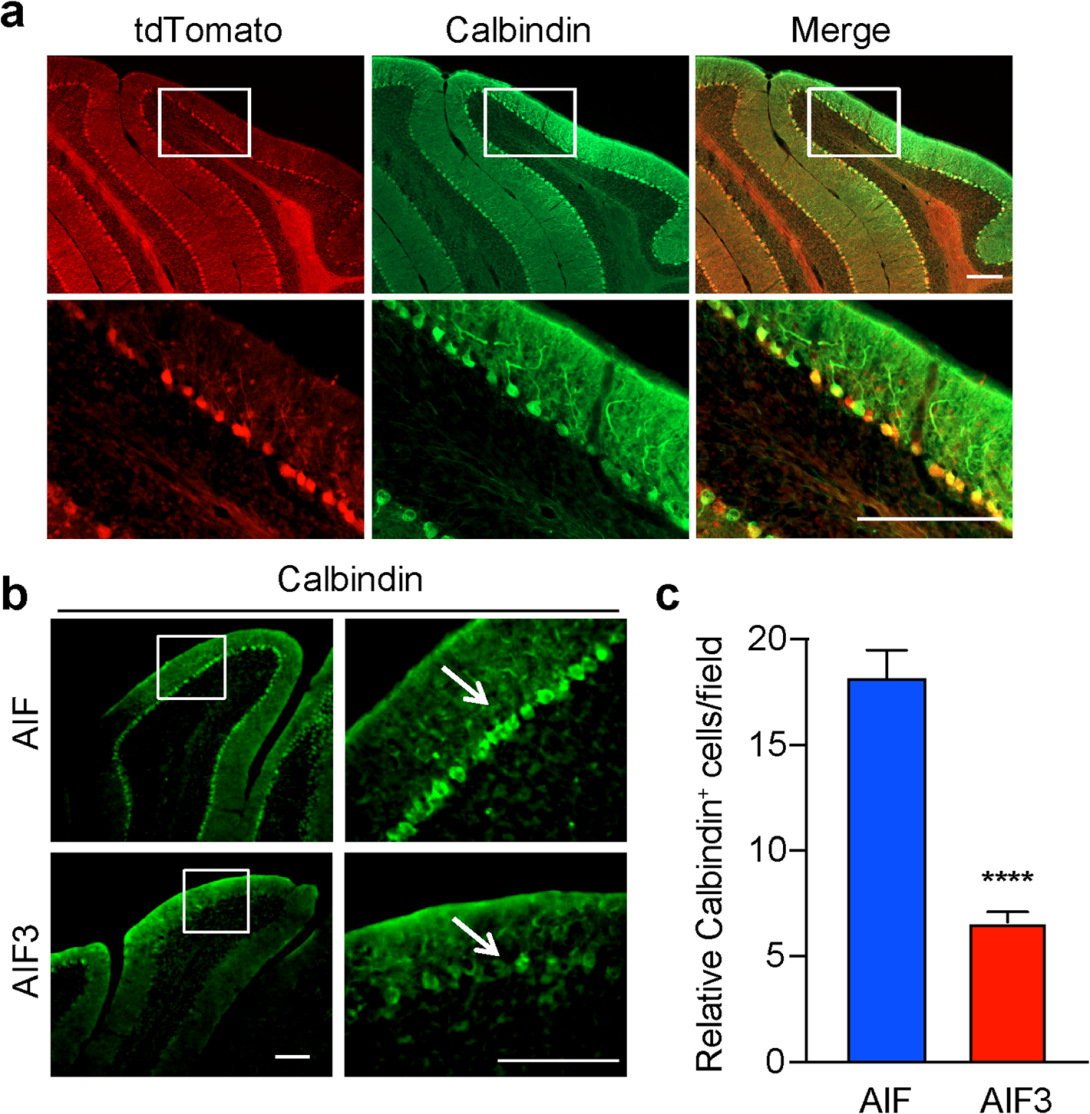
AIF3 induced neurodegeneration of Purkinje cells in cerebellum. **a** Expression of CamKIIα-iCre in Purkinje cells indicated by td-Tomato. CamKIIα-iCre mouse was crossed with a Cre reporter Td-Ai14 mouse. The expression of iCre was indicated by td-Tomato and Purkinje cells indicated by Calbindin in 6-month-old mouse. Top panel: overview; Bottom panel: magnification of the box region. Scale bar: 200 μM. **b** Calbindin immunostaining in 3-month-old AIF3 splicing mice (AIF^fl/Y^/CamKIIα-iCre+) and littermate control AIF mice (AIF^fl/Y^/CamKIIα-iCre-). The arrows indicate calbindin-positive Purkinje cells. Scale bar, 200 μm. Representative images are from at least three independent experiments. **c** Quantification of the relative Purkinje neuron number per field in the cerebellum of AIF and AIF3 splicing mice. Data are shown as mean ± S.E.M. and analyzed for statistical significance by Student *t* test. *n* = 5. ****p < 0.0001

Next, we characterized AIF3 splicing mouse and found that ventricles were enlarged in AIF3 splicing mouse brain compared to its littermate control at P90 (Fig. 4a). Substantial neurodegeneration was observed in the forebrain of AIF3 splicing mice, especially in the somatosensory cortex (Fig. 4b, i-i’ & c), piriform cortex (Fig. 4b, ii-ii’ & c), thalamus (Fig. 4b, iii-iii’ & c), and hippocampus (Fig. 4b, iv-vii, iv’-vii’ & c). In contrast, no granule cell loss was observed in the cerebellum (Fig. 4b, viii, viii’ & c). CamKIIα is highly expressed in Purkinje cells in the cerebellum in addition to the forebrain [40]. Consistently, expression of AIF3 significantly reduced the number of calbindin-positive Purkinje cells in 3-month-old AIF3 splicing mice (Fig. 5). Taken together, these data indicate that AIF3 splicing switch leads to neurodegeneration in the mouse brain.

**Fig. 6:**
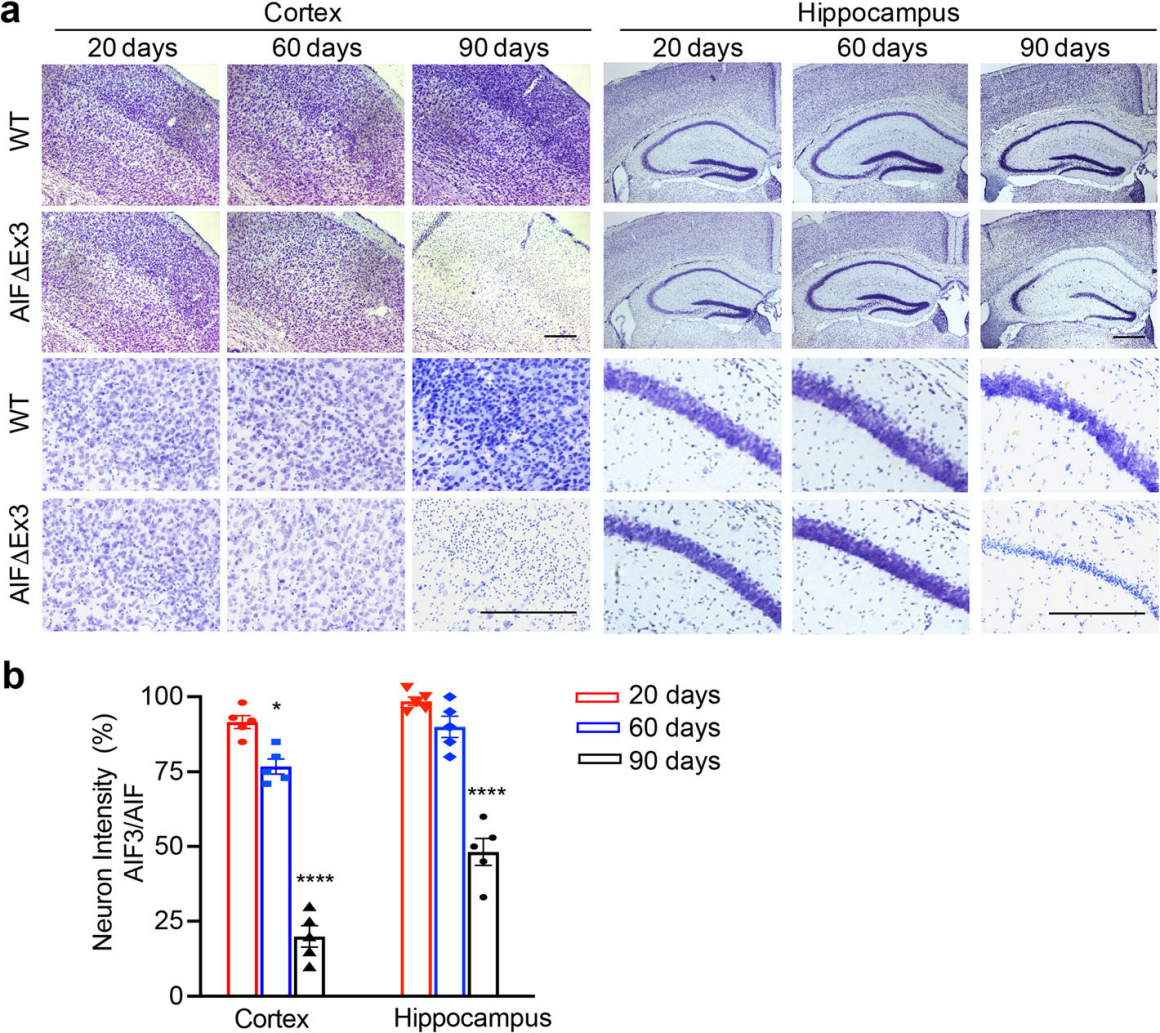
AIF3 splicing caused progressive neurodegeneration in the forebrain. **a** Representative Nissl staining images of cortex and hippocampus in AIF3 splicing mice (AIF^fl/Y^/CamKIIα-iCre+) and littermate control AIF mice (AIF^fl/Y^/CamKIIα-iCre-) at P20, P60 and P90 days. Scale bar, 200 μm. **b** Quantification of neuron loss regions in AIF3 splicing cortex and hippocampus vs. WT cortex and hippocampus respectively at P20, P60 and P90. Data are shown as mean ± S.E.M Data are shown as mean ± S.E.M and analyzed for statistical significance by one-way ANOVA. *n* = 5. ****p < 0.0001; *p < 0.05

**Fig. 7:**
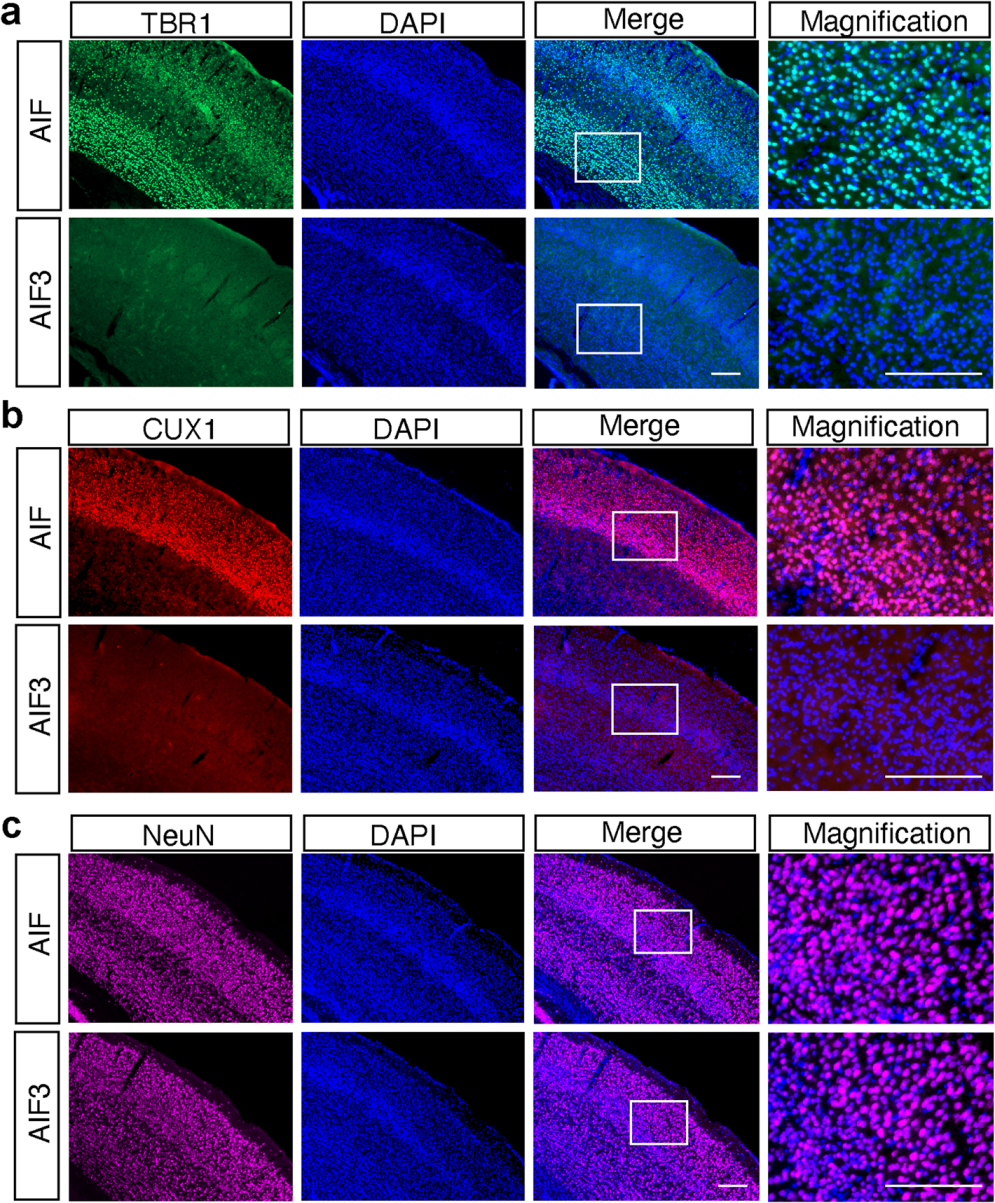
Characterization of neuron loss in the cortex of AIF3 splicing mice at P60. **a** Representative images of TBR1^+^ neurons in AIF3 splicing mice (AIF^fl/Y^/CamKIIα-iCre+) and littermate control AIF mice (AIF^fl/Y^/CamKIIα-iCre-) at P60. Scale bar, 200 μm. **b** Representative images of CUX1^+^ neurons in AIF3 splicing mice (AIF^fl/Y^/CamKIIα-iCre+) and littermate control AIF mice (AIF^fl/Y^/CamKIIα-iCre-) at P60. Scale bar, 200 μm. **c** Representative images of NeuN^+^ neurons in AIF3 splicing mice (AIF^fl/Y^/CamKIIα-iCre+) and littermate control AIF mice (AIF^fl/Y^/CamKIIα-iCre-) at P60. Scale bar, 200 μm. Representative images are from at least three independent experiments

*CamKIIα* gene expression is negligible until birth and increased 10-fold during P1-P21 and an additional 2.5-fold increase by P90 [31]. Thus, we compared the effect of AIF3 splicing switch on neuron loss at P20, P60 and P90 by Nissl staining. No obvious neuron loss was observed in the cortex or hippocampus of AIF3 splicing mice at P20, similar to WT littermates (Fig. 6a, b). At P60, partial neuron loss was observed in the cortex (25%), but not hippocampus, of AIF3 splicing mice (Fig. 6a, b). In contrast, there was substantial neuron loss in the somatosensory cortex (80%) and CA1 and CA3 regions of the hippocampus (48%) in AIF3 splicing mice at P90 (Fig. 6a, b). To further determine if there are specific types of neuron loss at P30 and P60, immunostaining using cortex layer markers TBR1 (mainly in layer VI), CUX1 (mainly in Layer II-III) and NeuN (general neuron marker), was performed. At P30, the expression of TBR1 and CUX1 was observed in both AIF3 splicing mice and its littermate controls (Supplementary Figure 3). The difference if any between AIF3 splicing mice and its littermate controls was not obvious. Remarkably, we found that the expression of TBR1 and CUX1 was almost completely eliminated from AIF3 cortex layers at P60 (Fig. 7a, b), although NeuN staining (Fig. 7c), DAPI staining (Fig. 7) and Nissl staining (Fig. 6) were only reduced moderately. These findings reveal that AIF3 splicing switch causes progressive neurodegeneration without obviously affecting neural differentiation process.

### Expression of AIF3 in cortical neurons causes neurotoxicity in vitro

**Fig. 8:**
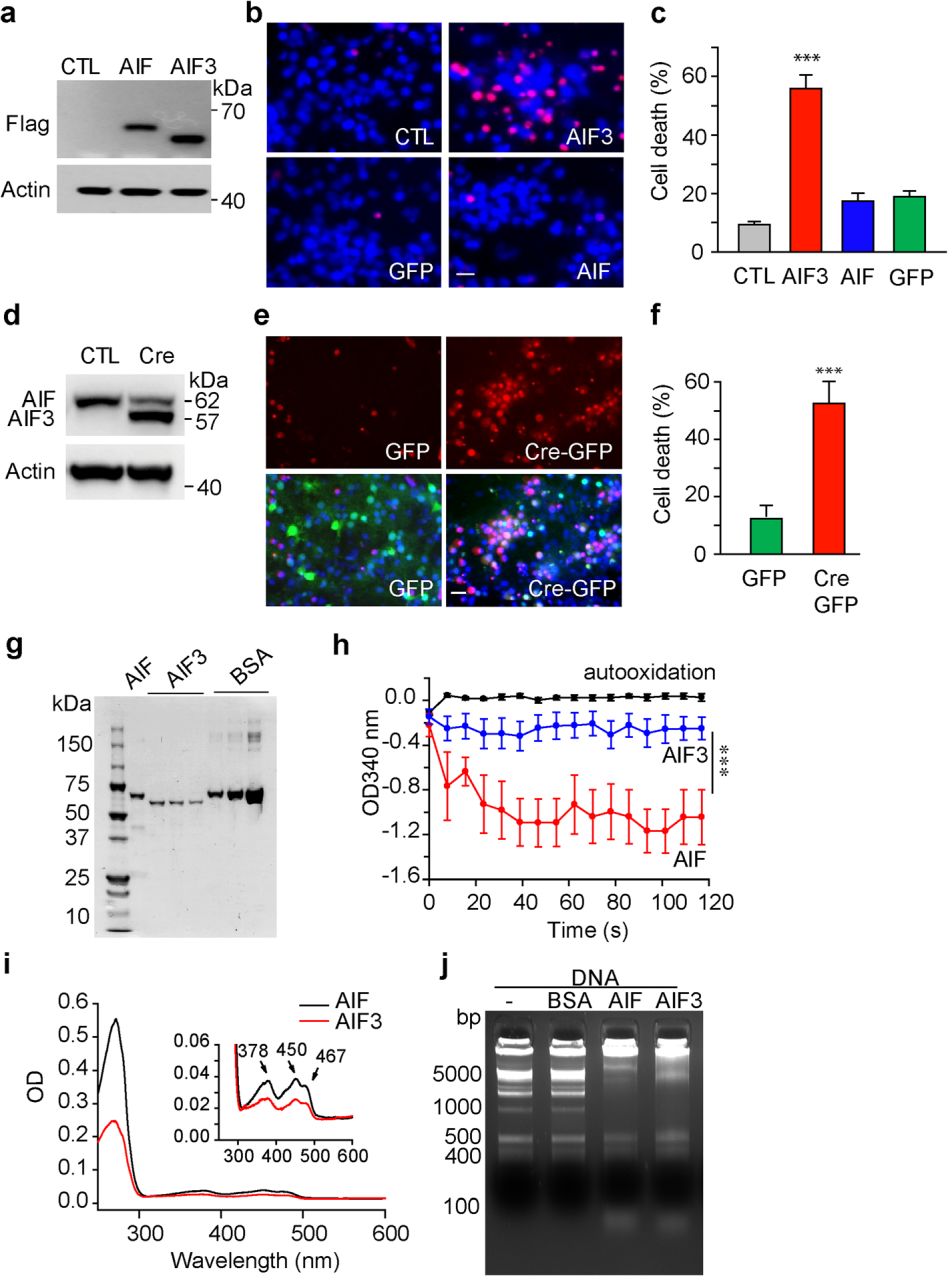
Characterization of AIF3 neurotoxicity effects in cortical neurons and its biochemical properties in vitro. **a** Expression of AIF and AIF3 in Hq cortical neurons 3 days after lentiviral transduction. **b** AIF3 expression in Hq cortical neurons caused neuronal death 4 days after lentiviral transduction as indicated by propidium iodide staining. Scale bar, 20 μm. **c** Quantification of AIF3-induced cytotoxicity in Hq cortical neurons 4 days after lentiviral transduction. Data were shown as mean ± S.E.M. *n* = 4. ***p < 0.001 by one-way ANOVA. **d** Expression of AIF3 in AIF fl/fl cortical neurons 3 days after GFP or Cre-GFP lentiviral infection (1.0 × 10^9^ T.U./ml). **e** AIF3 expression in AIF^fl/Y^/AIF^fl/fl^ cortical neurons caused neuronal death 5 days after GFP or CRE-GFP lentiviral infection as indicated by propidium iodide staining. Scale bar, 20 μm. **f** Quantification of AIF3-induced cytotoxicity in cortical neurons 5 days after GFP or CRE-GFP lentiviral infection. Data are shown as mean ± S.E.M. *n* = 4. ***p < 0.001 by Student *t* test. **g** AIF (62 kDa) and AIF3 protein purification. BSA was used as purity and quantity control. **h** The NADH oxidase activity of purified AIF (3 μM) and AIF3 (3 μM) was determined by monitoring changes in absorbance at OD340 nm. Data were shown as mean ± S.E.M. *n* = 3. ***p < 0.001 by two-way ANOVA. **i** FAD binding of purified AIF (1 μM) and AIF3 (1 μM) was determined by spectrophotometric wavelength scanning. **j** DNA retardation assay of purified AIF (3 μM) and AIF3 (3 μM) by incubating with 1 kb plus DNA ladder (200 ng) for 30 min. BSA was used as a negative control

To distinguish the actions of AIF and AIF3 in neurodegeneration, we prepared cortical neurons from Hq mice and infected cells with lentivirus carrying Flag-tagged AIF or AIF3. Both AIF and AIF3 protein were similarly expressed in Hq cortical neurons 3 days after lentiviral infection (Fig. 8a). AIF3 expression caused neuronal cell death at day 4 following lentiviral infection, whereas expression of AIF or GFP did not induce neurotoxicity (Fig. 8b, c). To further determine the role of endogenous AIF3, cortical neurons were prepared from E15.5 AIF^fl/Y^/AIF^fl/fl^ embryos and then infected with lentivirus carrying GFP or Cre-GFP (Fig. 8d). Three days after Cre-lentiviral transduction, AIF3 was successfully induced in neurons and AIF expression was decreased (Fig. 8d). Five days after Cre-GFP lentiviral transduction, over 50% of neurons died, while GFP-lentiviral transduced neurons exhibited minimal neuronal cell death (Fig. 8e, f). These data indicate that expression of AIF3 causes cytotoxicity in cultured neurons in vitro.

### AIF3 splicing switch mediates mitochondrial dysfunction in vitro and in vivo

AIF has flavin adenine dinucleotide (FAD)-binding and DNA-binding capabilities and also acts as an NADH oxidase in mitochondria [6]. GST-tagged AIF (62 kDa mature form) and AIF3 proteins were expressed and purified from bacteria, and then the GST tag was removed (Fig. 8g). NADH oxidase activity of purified AIF3 and AIF were compared by monitoring changes in absorbance in vitro. NADH oxidase activity of AIF3 (3 μM) was significantly lower than that of AIF at the same concentration (Fig. 8h). Both AIF and AIF3 had typical FAD-binding peaks at 378 nm, 450 nm and shoulder at 467 nm (Fig. 8i). However, the FAD-binding affinity of AIF3 (1 μM) was substantially reduced (Fig. 8i). Unlike their NADH oxidase activity and FAD-binding affinity, both AIF3 and AIF had a similar binding ability to DNA and caused DNA retardation (Fig. 8j). These data indicate that AIF3 has reduced NADH oxidase activity and FAD binding ability in vitro*,* but its DNA binding activity is retained.

**Fig. 9:**
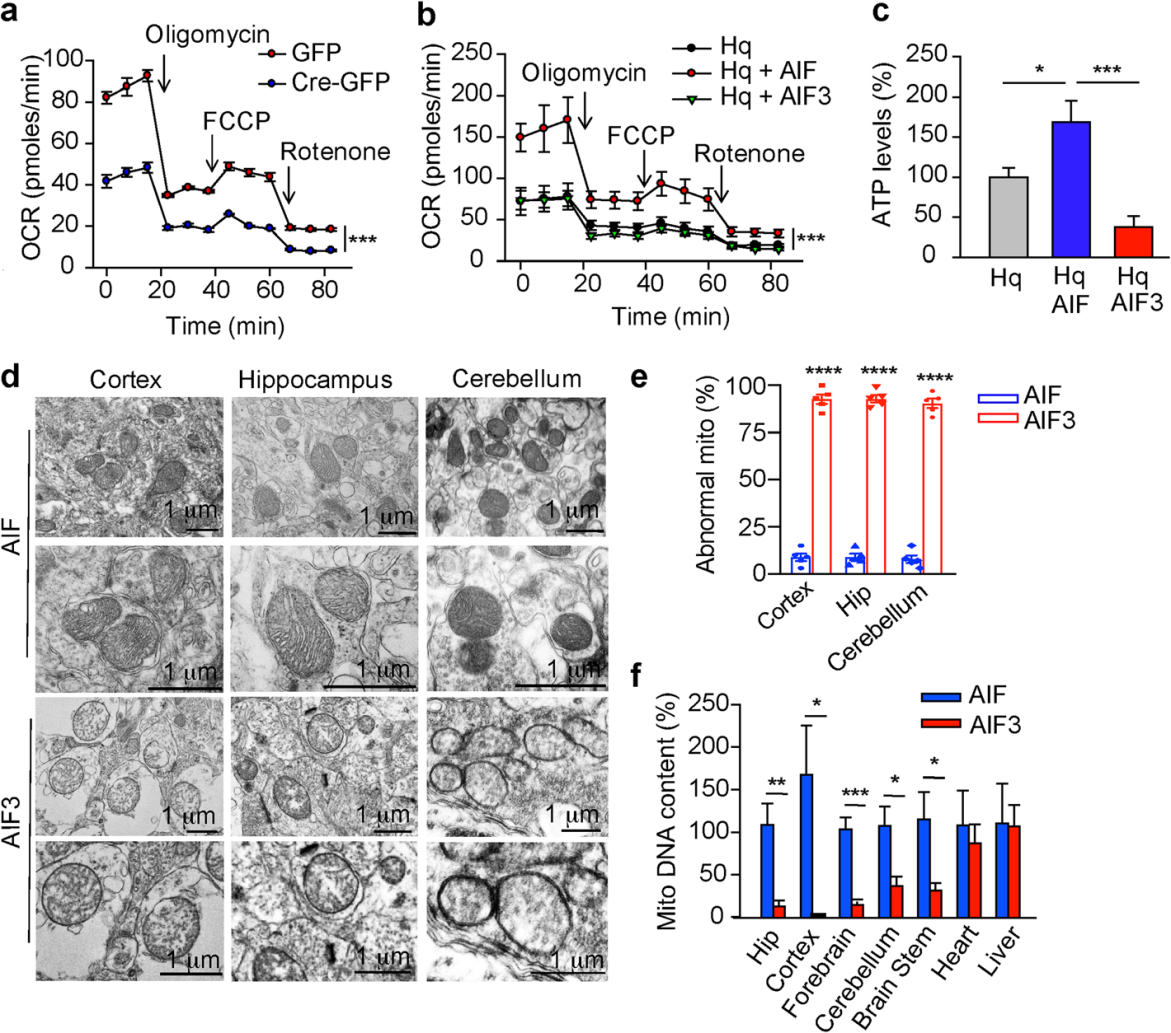
Effects of AIF and AIF3 expression on mitochondrial functions in vitro and in vivo. **a** OCR in AIF^fl/Y^/AIF^fl/fl^ cortical neurons 3 days after GFP or Cre-GFP lentivirus infection was measured after exposure to 1 μM oligomycin, 3 μM FCCP, and 1 μM rotenone. Data are shown as mean ± S.E.M. *n* = 3. ***p < 0.001 by two-way ANOVA. **b** OCR in Hq cortical neurons 3 days after AIF or AIF3 lentiviral infection was measured by Seahorse Bioscience XF24 Extracellular Flux Analyzer after exposure to 1 μM oligomycin, 3 μM FCCP, and 1 μM rotenone. Data are shown as mean ± S.E.M. *n* = 3. ***p < 0.001 by two-way ANOVA. **c** Intracellular ATP levels were measured in Hq cortical neurons 3 days after AIF or AIF3 lentiviral infection using a luminescence ATP assay kit. Data are shown as mean ± S.E.M. *n* = 3. *p < 0.05, ***p < 0.001. **d** Mitochondrial structure of neurons in the cortex, hippocampus and cerebellum of AIF3 splicing mice (AIF^fl/Y^/CamKIIα-iCre+) and their littermate control AIF mice (AIF^fl/Y^/CamKIIα-iCre-) by electron microscopy. **e** Quantification of abnormal mitochondria (mito) in the cortex, hippocampus (Hip) and cerebellum of AIF3 splicing mice and their littermate control AIF mice. Data are shown as mean ± S.E.M. *n* = 5. ****p < 0.0001 by one-way ANOVA. **f** AIF3 splicing reduced mitochondrial biogenesis in brain, but not heart or liver of AIF3 splicing mice. The ratio of mitochondrial DNA relative to nuclear genomic DNA was determined by quantitative real-time PCR using primers for cytochrome b (mitochondrial) and ribosomal protein S2 (RPS2, nuclear). The data observed in AIF3 splicing mice were normalized to the littermate control AIF mice. Data are shown as mean ± S.E.M. *n* = 3. ***p < 0.001, **p < 0.01, *p < 0.05 by one-way ANOVA

Oxidation of NADH in mitochondria is critical for ATP synthesis. To study the role of AIF3 splicing switch in mitochondrial respiration, cortical neurons were prepared from AIF^fl/Y^/AIF^fl/fl^ embryos and infected with Cre-GFP lentivirus. AIF3 splicing switch significantly decreased the basal oxygen consumption rate as compared to controls transduced with GFP lentivirus (Fig. 9a). Hq cortical neurons also had lower oxygen consumption rate due to loss-of-AIF (Fig. 9b). Expression of AIF in Hq cortical neurons significantly increased the basal oxygen consumption rate, whereas expression of AIF3 failed to rescue the basal oxygen consumption rate (Fig. 9b). Consistently, overexpression of AIF in Hq cortical neurons increased ATP production, whereas AIF3 expression in Hq cortical neurons did not rescue the ATP production (Fig. 9c). In contrast, it even significantly reduced ATP levels (Fig. 9c). These data suggest that AIF3 at least loses part of AIF functions and contributes to the dysregulation of mitochondrial oxygen consumption rate and ATP production.

To further study the role of AIF3 splicing switch in mitochondrial functions, we examined mitochondrial ultrastructure in AIF3 splicing mouse brain at P70 by electron microscopy. The majority of neurons in the cortex, hippocampus, and cerebellum of AIF3 splicing mice had severe defects in mitochondrial morphology and cristae organization (Fig. 9d, e). Mitochondria in AIF3 splicing mice were larger in diameter and exhibited vesiculation of their inner membranes, whereas littermate control mitochondria were intact and well organized (Fig. 9d, e). Mitochondrial DNA mass was significantly reduced in the forebrain, cerebellum, and brain stem of AIF3 splicing mice compared to littermate controls (Fig. 9f). No difference in mitochondrial mass was observed in heart and liver of AIF3 and AIF mice where AIF3 was not expressed (Fig. 9f; Supplementary Figure 1c). Taken together, our findings demonstrate that AIF3 splicing switch inhibits ATP production, oxygen consumption, and mitochondrial biogenesis, leading to mitochondrial dysfunction in mouse brain.

### AIF3 splicing switch promotes chromatin condensation

**Fig. 10:**
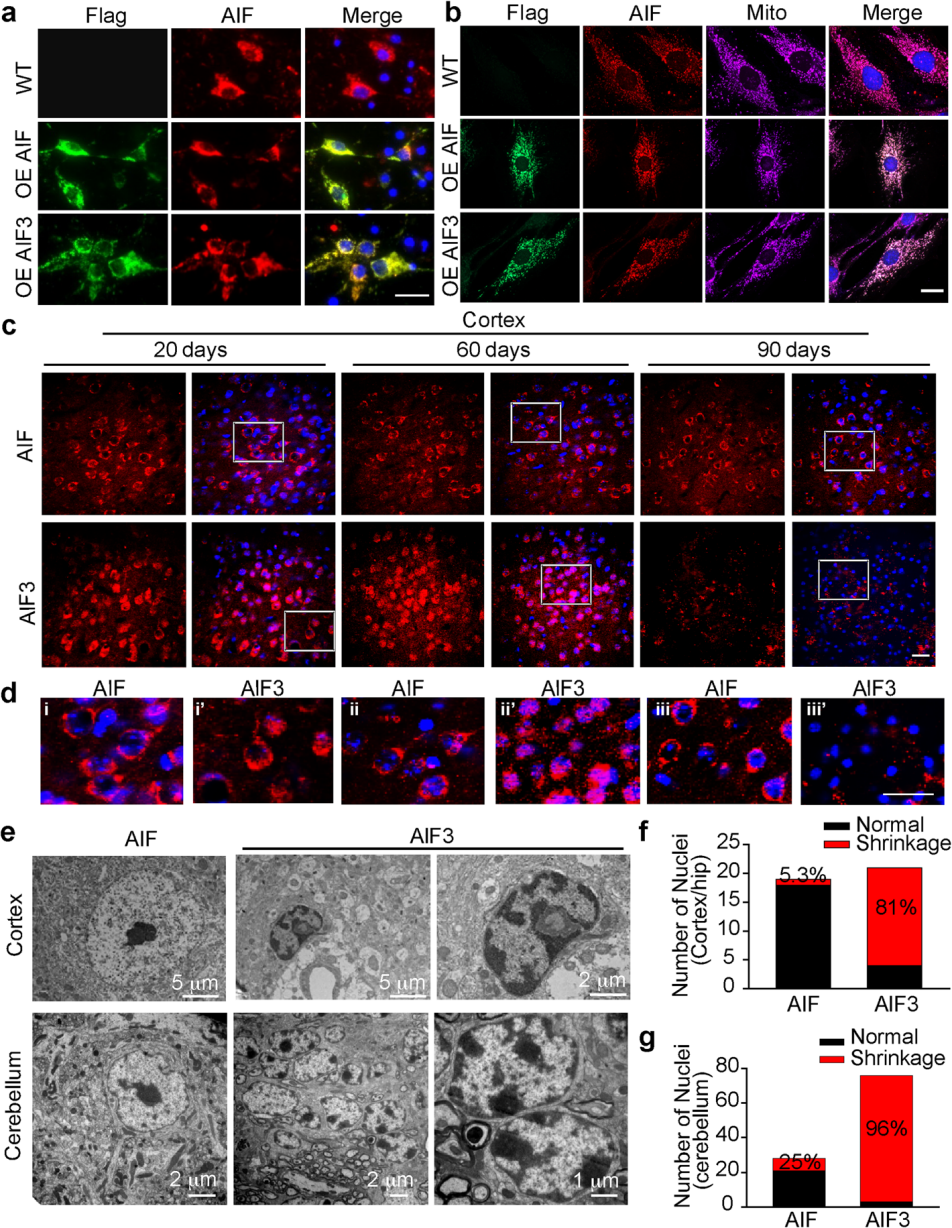
Expression of AIF3 increased chromatin condensation and nuclear shrinkage. **a** Immunostaining of Flag-tagged AIF and AIF3 and their colocalization with endogenous AIF in cortical neurons. OE, overexpression. Scale bar, 20 μm. **b** Immunostaining of Flag-tagged AIF and AIF3, endogenous AIF, and mitotracker (mito) in MEFs. OE, overexpression. Scale bar, 20 μm. Representative images are from at least three independent experiments. **c** Representative AIF3/AIF localization changes in the cortex of AIF3 splicing mice (AIF^fl/Y^/CamKIIα-iCre+) and littermate control (AIF^fl/Y^/CamKIIα-iCre-) AIF mice at P20, P60 and P90. Scale bar, 50 μm. **d** High magnification images for highlighted areas in **b**. i, ii, iii, AIF mice at P20, P60 and P90 respectively, and i’, ii”, iii”, AIF3 splicing mice at P20, P60 and P90 respectively. Scale bar, 50 μm. **e**, Representative images of nuclei of neurons in the cortex and cerebellum of AIF3 splicing mice (AIF^fl/Y^/CamKIIα-iCre+) and littermate control AIF mice (AIF^fl/Y^/CamKIIα-iCre-) by electron microscopy. **f** & **g** Quantification of nuclear shrinkage of neurons in the forebrain (**f**) and cerebellum (**g**) of AIF3 splicing mice and their littermate control AIF mice. Data are shown as mean. *n* = 4. Representative images are from at least three independent experiments

AIF is translocated to the nucleus to induce chromatin condensation and nuclear shrinkage, leading to cell death [28, 29]. The subcellular localization of AIF3 was studied by immunochemical and MitoTracker staining. We showed that exogenous Flag-AIF and Flag-AIF3 were colocalized with endogenous AIF in cortical neurons (Fig. 10a). Likewise, AIF3 was localized at mitochondria as indicated by MitoTracker and colocalized with endogenous AIF in MEFs, where expression of AIF3 did not obviously alter mitochondrial size or morphology (Fig. 10b). In vivo immunohistochemistry of the cortex further showed that AIF3 was localized at mitochondria, and only a small portion of AIF3 was observed in the nucleus at P20. In contrast, the majority of AIF3 was translocated into the nucleus at P60 (Fig. 10c, d). At P90, little AIF3 staining was detected in the cortex of AIF3 splicing mice due to severe neuron loss (Fig. 10c, d). Distinct from AIF3, AIF was primarily found at mitochondria from P20 to P90 (Fig. 10c, d). Electron microscopy revealed that approximately 81% of nuclei in the cortex of AIF3 splicing mice displayed chromatin condensation and nuclear shrinkage, which was significantly higher than that in the littermates (5.3%) (Fig. 10e, f). Similar phenotypes were also observed in the cerebellum of AIF3 splicing mice (Fig. 10e, g). Taken together, these data indicate that AIF3 is prone to translocate to the nucleus and promote chromatin condensation and nuclear shrinkage, thereby increasing neuronal susceptibility to death in vivo.

### The synergistic effect of loss-of-AIF and gain-of-AIF3 contributes to AIF3 splicing switch-induced neurodegeneration in vivo

**Fig. 11:**
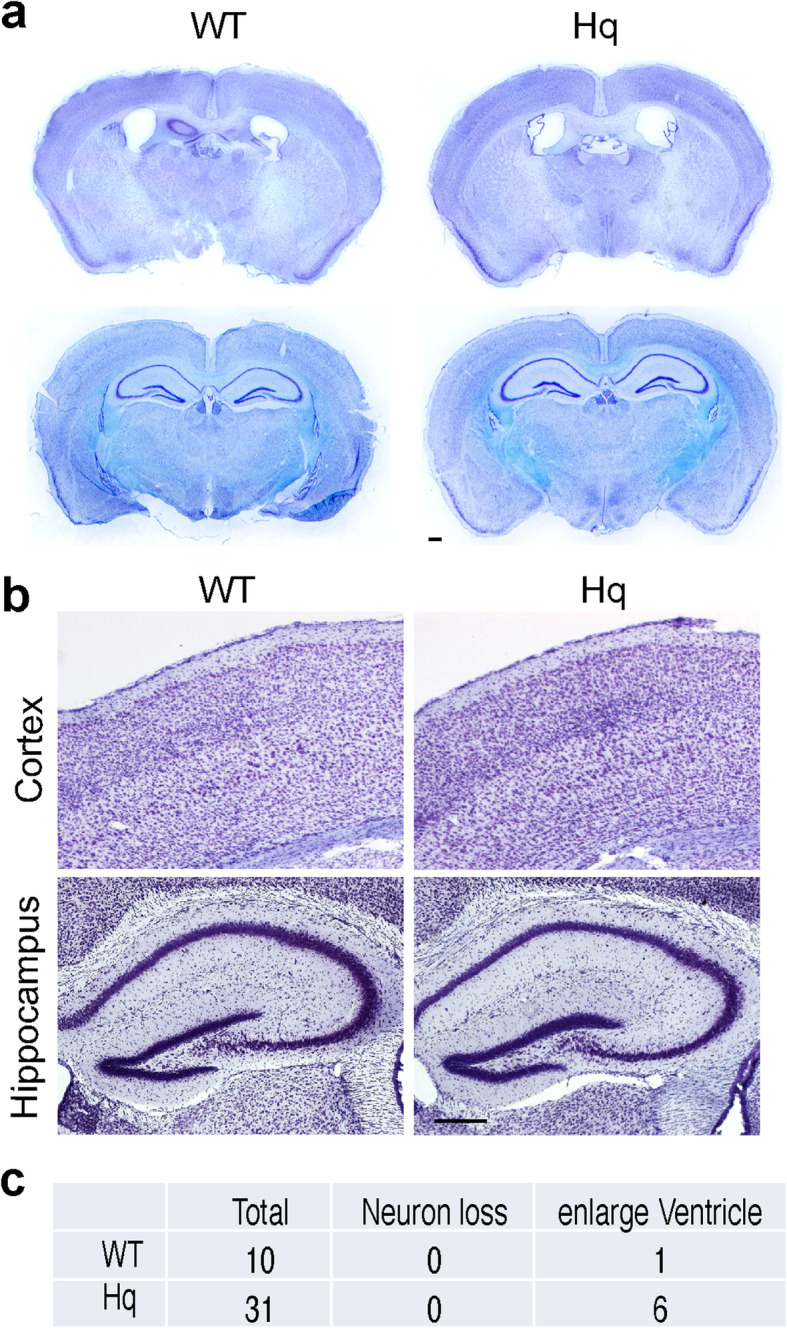
Loss of AIF in Hq mice did not obviously cause neurodegeneration in the forebrain. **a** Representative overview images of Hq mouse brain (P100), including somatosensory cortex, piriform cortex, thalamus and hippocampus. Nissl staining images from C57BL/6 J-A^w-J^/J mice with the same genetic background were used as the control. Scale bar, 200 μm. **b** Nissl staining of somatosensory cortex and hippocampus from Hq and their control mice at P100. Scale bar, 200 μm. **c** Quantification of mouse number with either obvious neuron loss or enlarge ventricle

AIF3 splicing switch from AIF to AIF3 involves both gain-of-AIF3 and loss-of-AIF events. We next dissected whether loss-of-AIF, gain-of-AIF3, or both contributes to neurodegeneration in vivo. To study whether loss-of-AIF mainly contributes to neurodegeneration observed in AIF3 splicing mice, we used Hq mice as a model as conditional deletion of AIF in mouse brain is embryonic lethal and Hq mice have 80% of AIF reduction (Fig. 1, Supplementary Figure 1b, c, e). We found that no obvious neuron loss was detected in the cortex or hippocampus of male Hq mice at P100 (Fig.11a, b), despite Purkinje neuron loss in the cerebellum of 6-month-old Hq mice reported previously [10, 26, 41]. However, 19.4% of Hq mice showed enlarged ventricle (Fig. 11c), which was a feature observed in AIF3 splicing mice. Hq hemizygous males and homozygous females showed a smaller body weight at 3–6 months old [10, 41]. These data suggest that loss-of-AIF is necessary, but not sufficient, to cause robust neurodegeneration observed in AIF3 splicing mice.

**Fig. 12:**
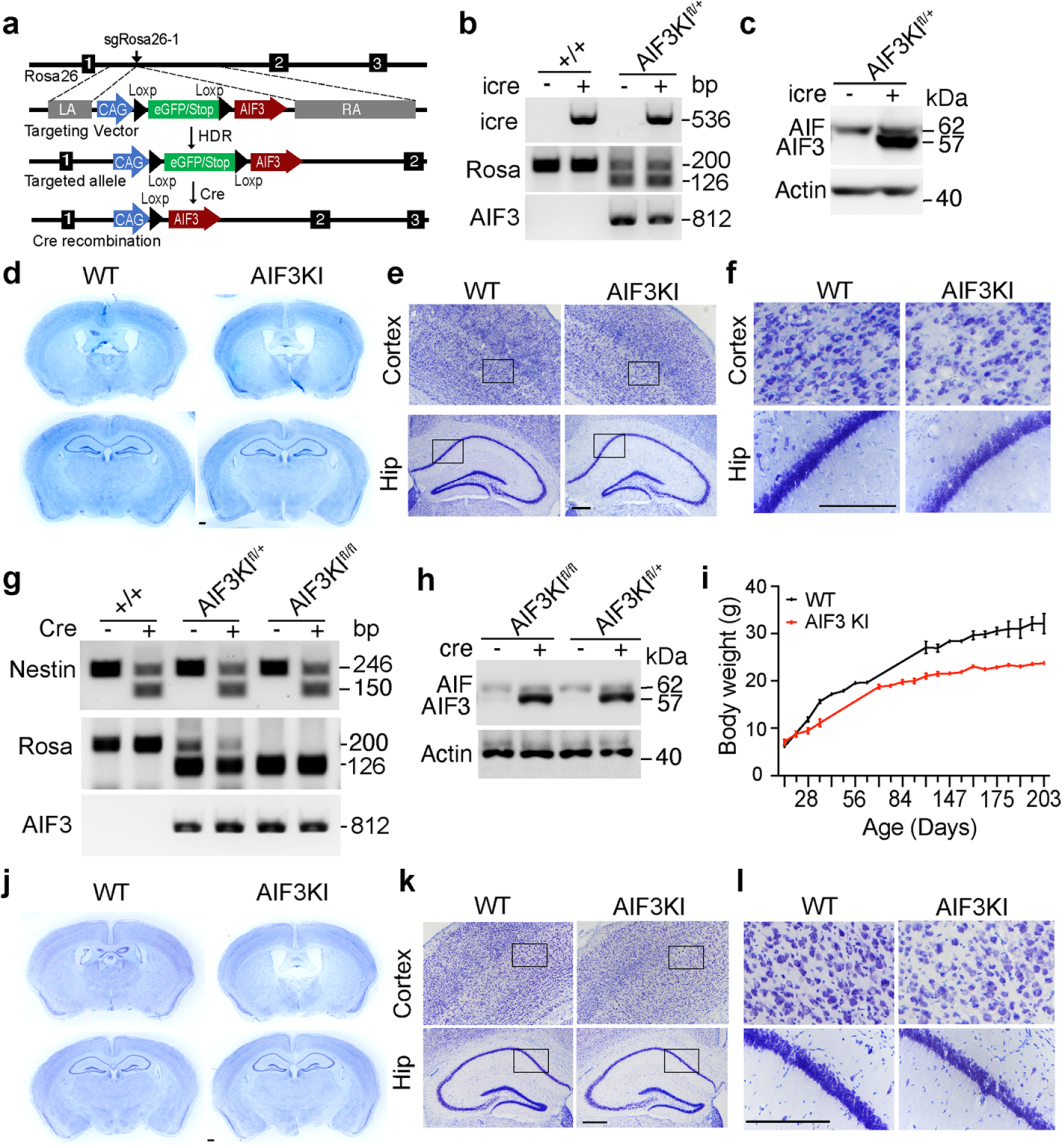
Characterization of AIF3KI mouse model and its role in neurodegeneration in the forebrain. **a** Strategy for insertion of the LoxP-eGFP/STOP-LoxP-AIF3 (LSL-AIF3) cassette into the mouse Rosa26 locus. sgRosa26–1 and Cas9 introduce a double-strand break between 1 kb and 4 kb fragments used as homology arms in the targeting vector. Homology-directed repair (HDR) leads to the insertion of the cassette. Cre recombination induces C-terminal Flag-tagged AIF3 expression. LA, left arm. RA, right arm. **b** PCR analysis of CamKIIα-iCre recombination at the Rosa26 locus in 7-day-WT and AIF3KI male and female mice (PCR ID: iCre, Rosa, AIF3, Table 2). **c** Expression of AIF3 in the cortex of male WT (AIF3KI^fl/+^, CamkII-iCre-) and AIF3KI (AIF3KI^fl/+^, CamkII-iCre+) at P105 determined by E1-AIF antibody. **d** Nissl staining overview of WT (AIF3KI^fl/+^, CamkII-iCre-) and AIF3KI (AIF3KI^fl/+^, CamkII-iCre+) male mice at P105. **e-f** Nissl staining of cortex and hippocampus from WT (AIF3KI^fl/+^, CamkII-iCre-) and AIF3KI (AIF3KI^fl/+^, CamkII-iCre+) male mice at P105. Box indicates the zoom in images shown in **f**. Scale bar, 200 μm. *n* = 3. **g** PCR analysis of nestin-Cre recombination at the Rosa26 locus in 7-day-WT and AIF3KI male and female mice (PCR ID: nestin-Cre, Rosa, AIF3, Table 2). **h** Expression of AIF3 in the cortex of male WT (AIF3KI^fl/+^, nestin-Cre-), and AIF3KI (AIF3KI^fl/fl^, nestin-Cre + and AIF3KI^fl/+^, nestin-Cre+) at P83 determined by E1-AIF antibody. **i** Measurement of the body weight of AIF3KI male mice (AIF3KI^fl/fl^, nestin-Cre+, *n* = 17) and their littermate control mice (AIF3KI^fl/fl^, nestin-Cre-, *n* = 12). Data are shown as mean ± S.E.M. and analyzed for statistical significance by two-way ANOVA. *** p < 0.001. **j** Nissl staining overview of WT (AIF3KI^fl/fl^, nestin-Cre-) and AIF3KI (AIF3KI^fl/fl^, nestin-Cre+) male mice at P83. **k-l** Nissl staining of cortex and hippocampus from WT (AIF3KI^fl/fl^, nestin-Cre-) and AIF3KI (AIF3KI^fl/fl^, nestin-Cre+) male mice at P83. Box indicates the zoom in images shown in **l**. Scale bar, 200 μm

To explore the role of gain-of-AIF3 in neurodegeneration in the presence of AIF, we established a conditional AIF3KI mouse model by inserting the loxP-stop-loxP-AIF3 (LSL-AIF3) sequence into Rosa26 locus using CRISPR/Cas9 technique and crossed these mice with CamkIIα-iCre mice (Fig. 12a, b, c). Expression of heterozygous AIF3 in CamkIIα-iCre+ mice was induced in the forebrain (Fig. 12c). Both male and female AIF3KI mice appeared normal for at least 7 months. No obvious neuron loss was observed in the cortex and hippocampus of AIF3KI mice at P105 (Fig. 12d). However, the lateral ventricles in AIF3KI mice were enlarged (Fig. 12d). Moreover, the neuronal cell density was reduced in both cortex and hippocampus (Fig. 12e, f). The consistent results were also found in another AIF3 KI mouse model expressing neuronal cell-specific nestin-Cre (Fig. 12g, h). AIF3KI mice with nestin-Cre+ had a smaller body weight but survived for at least 7 months (Fig. 12i). Although enlarged lateral ventricles and reduced neuron density were detected in the cortex layer and hippocampus of AIF3 KI mice (AIF3 KI/nestin-Cre+), no substantial neuron loss was observed from the overview of AIF3KI mice at P83, distinct to what we had observed from AIF3 splicing mice (Fig. 12j, k, l), indicating that gain-of-AIF3 alone is not sufficient to cause severe neurodegeneration in vivo. Together, these data suggest that the synergistic effects of loss-of-AIF and gain-of-AIF3 trigger robust neurodegeneration in AIF3 splicing mice.

## Discussion

Here we identified a novel AIF3 isoform, which lacks the hydrophobic domain encoded by exons 2 and 3 of the *Aifm1* gene. AIF3 is a nature splicing isoform in C57BL/6 mouse brain containing 14 exons and is distinct from five other known AIF isoforms identified by RACE using human kidney cDNA library [2, 3]. Although AIF3 is not detectable under physiological conditions, our studies reveal that AIF3 is a disease-inducible splicing isoform produced from the endogenous *Aifm1* gene. AIF3 expression is induced in the cortex of human postmortem brain with hypoxic-ischemic or hemorrhage stroke and mouse brain following ischemic stroke. However, AIF3 expression cannot be induced by hypoxia or OGD in cortical neuron cultures. Glial cells in vivo not only provide structural support but also regulate the release of gliotransmitters, neuroinflammation and metabolism [42]. Therefore, it might be due to the fact that in vitro neuron cultures lack neuron-glia interactions and cannot mimic the hypoxic-ischemic environment in brain. Interestingly, we showed that AIF3 splicing is robustly induced in mouse brain when exon 3 of the *Aifm1* gene is deleted, suggesting that exon 3 of the *Aifm1* gene is critical for AIF3 splicing regulation. Mouse with *Aifm1* exon 3 deletion appears to be a valuable model to understand biological functions of AIF3 splicing in brain.

Another key finding of this study is that AIF3 splicing switch in mouse brain causes neurodegeneration. AIF3 splicing mice display an early onset of aging phenotypes supported by our observations of neuron loss, enlarged ventricles, and alerted animal behavior at young ages. All AIF3 splicing mice eventually die between 2 to 4 months after birth. Given that AIF3 is induced in patients following hypoxic-ischemic brain injury, AIF3 splicing in these patients may trigger neurodegeneration. The Stroke-Dementia Association showed that about 10% of stroke patients develop post-stroke-neurodegeneration and signs of dementia within 1 year and stroke has been recognized as the second most common cause of dementia [43, 44]. In future studies, a tamoxifen inducible CamkIIα-CreERT mouse line might be used to induce comparable levels of AIF3 splicing observed in hypoxic-ischemic brain and directly study its effect on hypoxic-ischemic brain injury, post-stroke-associated neurodegeneration as well as other mitochondrial dysfunction-associated neurodegenerative diseases [45].

Hq and AIF3KI mice are valuable models allowing us to directly dissect the importance of loss-of-AIF and gain-of-AIF3 in neurodegeneration. We found that neither loss-of-AIF nor gain-of-AIF3 alone is sufficient to cause robust neurodegeneration as observed in AIF3 splicing mice at similar ages, although loss-of-AIF in aged Hq mice causing neurodegeneration in the cerebellum has been well appreciated [10, 26, 41]. The less obvious neurodegeneration effects in forebrain of Hq mouse might be partially due to the fact that Hq mice still have a small portion of AIF (20%) supporting its basic functions in each single cell, whereas AIF3 splicing mice completely switch AIF expression to AIF3 in CamkIIα-iCRE expressing cells. Since AIF knockout and telencephalon-specific AIF knockout both are embryonic lethal, this possibility cannot be excluded. On the other hand, it is also no doubt that gain-of-AIF3 contributes to mild neurodegeneration, which is supported by the observation of enlarged ventricle and mild neuron reduction in the forebrain. Moreover, gain-of-AIF3 in cultured Hq cortical neurons leads to neurotoxicity. Taken together, our three animal models provide the direct evidence that the synergistic effects of loss-of-AIF and gain-of-AIF3 contribute to neurodegeneration in AIF3 splicing mice.

Interestingly, AIF3 splicing switch in MEFs prepared from AIF^fl/fl^/AIF^fl/Y^ mice does not display obvious cytotoxicity, which is different from that in AIF3-overexpressed Hq cortical neurons. These AIF3 splicing MEFs even grow faster than WT MEFs with obvious increase in extracellular acidification, indicating altered cellular metabolism may overcome cytotoxicity in AIF3 splicing MEFs.

Mitochondrial dysfunction has been often linked to neurodegeneration, although the precise mechanism is less clear. AIF plays a pivotal role in mitochondrial bioenergetics that is critical for cell survival [26, 46, 47]. Decreased mitochondrial oxidative phosphorylation is a common feature observed in different AIF-deficient mouse models [26, 46, 47], supporting that mitochondrial defects in AIF3 splicing mice is at least partially caused by the loss-of-AIF, as AIF3 lacks NADH oxidase activity and FAD-binding affinity. However, loss-of-AIF alone is not sufficient to fully phenocopy mitochondrial dysfunction observed in AIF3 splicing mice as the effects of loss-of-AIF on mitochondrial structural alteration in various animal models are quite different from that in AIF3 splicing mice. For example, loss-of-AIF in telencephalon alters its mitochondrial cristae but does not disturb the overall mitochondrial morphology [24] and deletion of AIF in liver and muscle fails to obviously alter mitochondria size, distribution or ultrastructural structure [47]. AIF3 splicing mice showed the disorganization of overall mitochondrial structures with the larger diameter and vesiculation of their inner membranes. In line with this, overexpression of AIF3 in Hq cortical neurons reduces ATP production, supporting the notion that AIF3 gains additional detrimental functions to promote mitochondrial dysfunction.

AIF has been recognized as a key cell death mediator of parthanatos (caspase-independent cell death) when AIF is released from mitochondria and translocated to the nucleus under pathological conditions, especially ischemic brain injury [6, 27–29]. It eventually causes chromatin condensation and cell death [6, 27–29]. AIF3 lacks the hydrophobic domain and is expected to be easily released from mitochondria and translocated to nucleus. Indeed, our studies showed increased AIF3 nuclear translocation, chromatin condensation, and neuronal cell death in a time dependent manner in AIF3 splicing mice. Together, these findings uncover that both mitochondrial dysfunction and AIF3 nuclear translocation resulting from the synergistic effects of loss-of-AIF and gain-of-AIF3 contribute to AIF3 splicing-triggered neurodegeneration.

## Conclusions

We identified a novel disease-inducible AIF splicing isoform AIF3 in brain and established a conditional inducible AIF3 splicing mouse model. AIF3 splicing switch in brain plays a detrimental role in neurodegeneration and the underlying mechanism involves mitochondrial dysfunction and AIF3 nuclear translocation-triggered neuronal cell death, which is attributed to the synergistic effects of loss-of-AIF and gain-of-AIF3. The discovery of AIF3 isoform and establishment of AIF3 splicing mouse model may provide a valuable tool to understand the role of AIF3 splicing in aged brain and a potential therapeutic target to prevent or delay the progress of neurodegenerative diseases.

## Supplementary Information

**Additional file 1: Supplementary Figure 1**. Identification of AIF3 isoform.

**Additional file 2: Supplementary Figure 2**. Expression of AIF3 in neurons in vitro and mouse cortex under different pathological conditions. **Supplementary Figure 3.** Characterization of neuron marker expression in the cortex of AIF3 splicing mice at P30.

## Data Availability

The datasets used and/or analyzed during the current study are included in this article and its supplementary information files. The datasets are available from the corresponding authors on reasonable request.

## Abbreviations

AIF: Apoptosis-inducing factor
ATP: Adenosine triphosphate
FAD: Flavin adenine dinucleotide
fl: Floxed
Hq: Harlequin
KI: Knockin
MCAO: Middle Cerebral Artery Occlusion
MEFs: Mouse embryonic fibroblasts
MEM: Modified Eagle’s medium
PARP-1: Poly(ADP-ribose) polymerase 1
PCR: Polymerase chain reaction
RACE: Rapid amplification of cDNA end analysis
TTC: Triphenyl tetrazolium chloride
