# Supplementary material for: AIF3 splicing switch triggers neurodegeneration
Source: Mol Neurodegener. 2021 Apr 14;16:25. doi: 10.1186/s13024-021-00442-7 (PMC8048367; doi:10.1186/s13024-021-00442-7)
Supplement: Supplementary file 1 — Additional file 1: Supplementary Figure 1. Identification of AIF3 isoform. [file 13024_2021_442_MOESM1_ESM.pdf]

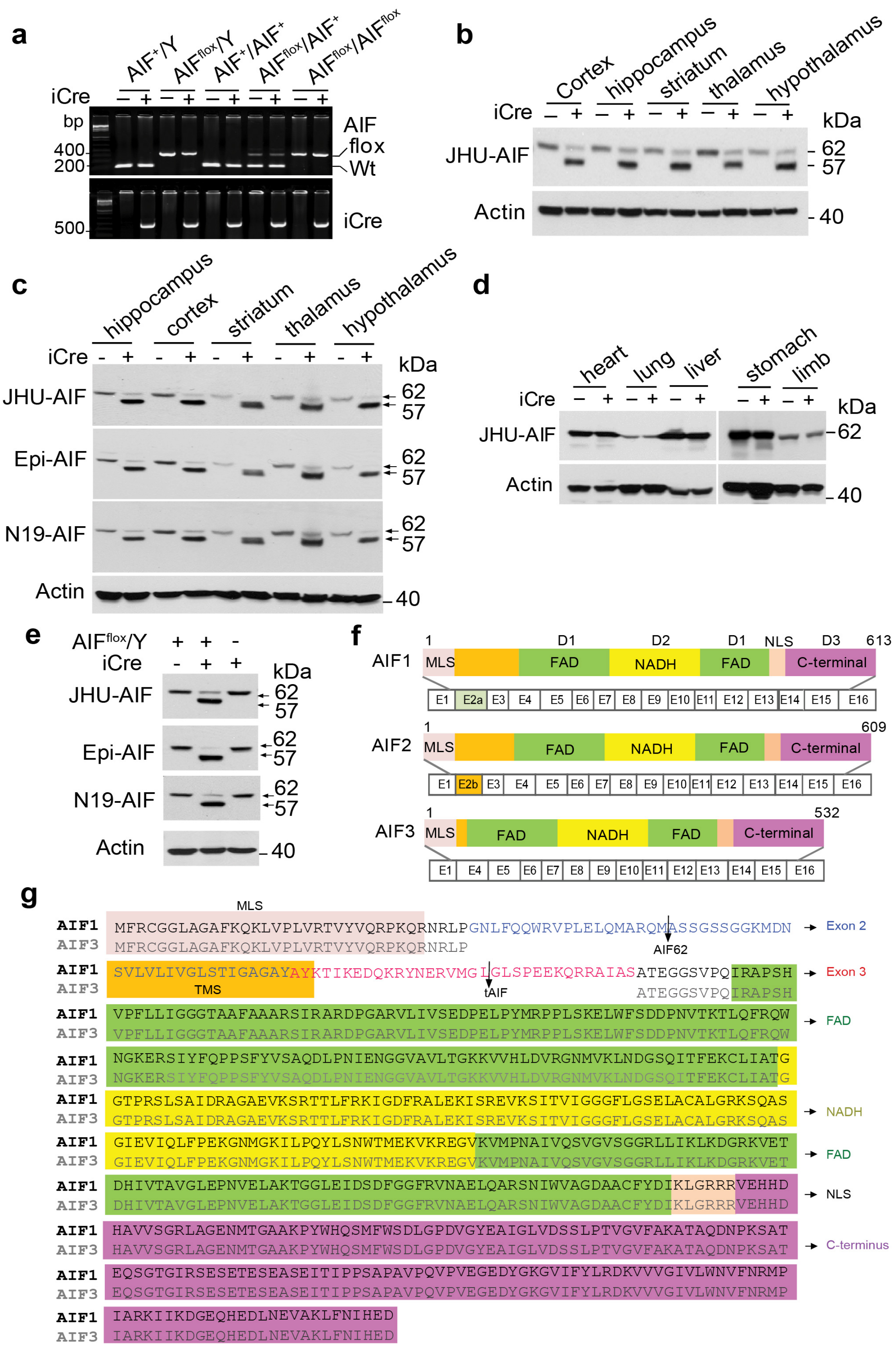

Supplementary Figure 1. Identification of AIF3 isoform. a, PCR analysis of CamKIIα-iCre

recombination at the AIF locus in 7-day-old $AIF^{fl/Y}$ or $AIF^{fl/fl}$ mice (PCR ID: AIFgeno and iCre, Table 2). **b,** Expression of AIF3 in different brain regions from $AIF^{fl/Y}$ mice with or without CamKIIα-iCre recombination at P20 determined by JHU-532 AIF antibody. **c** & **d,** Expression of AIF3 in different brain regions (**c**) and other organs (**d**) from $AIF^{fl/Y}$ mice with or without CamKIIα-iCre recombination at P70 determined by different AIF antibodies. **e**, No AIF3 was expressed in CamKIIα-iCre control mice at P70 determined by 3 different AIF antibodies. **f**, Scheme of the novel AIF3 isoform. AIF3 contains 14 exons skipping exons 2 and 3. **g,** Protein sequence and functional domains of mouse AIF3.
