# Supplementary material for: AIF3 splicing switch triggers neurodegeneration
Source: Mol Neurodegener. 2021 Apr 14;16:25. doi: 10.1186/s13024-021-00442-7 (PMC8048367; doi:10.1186/s13024-021-00442-7)
Supplement: Supplementary file 2 — Additional file 2: Supplementary Figure 2. Expression of AIF3 in neurons in vitro and mouse cortex under different pathological conditions. Supplementary Figure 3. Characterization of neuron marker expression in the cortex of AIF3 splicing mice at P30. [file 13024_2021_442_MOESM2_ESM.pdf]

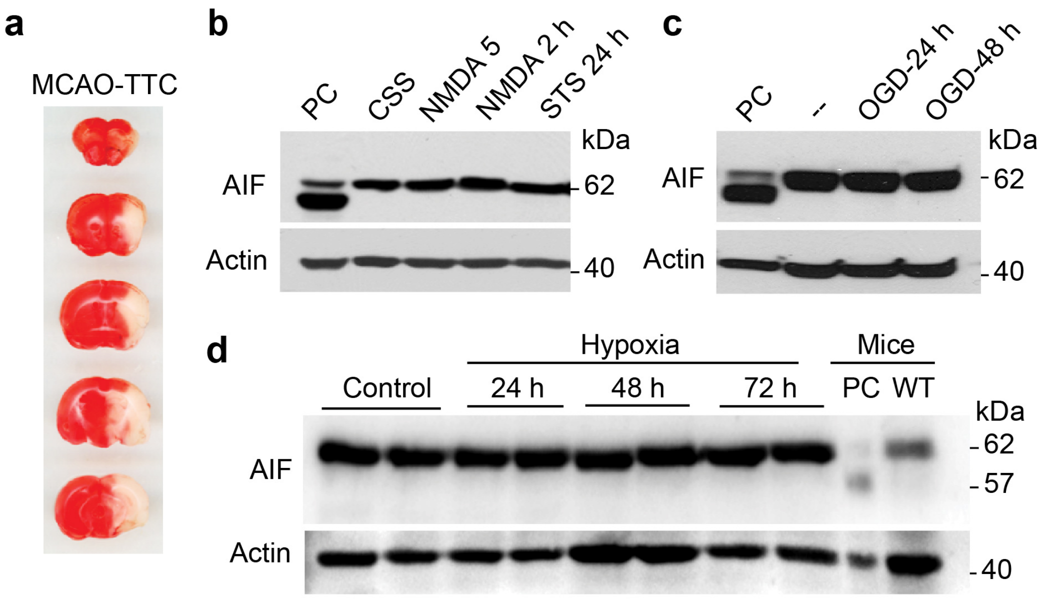

e

Id	Age	Sex	Diagnosis	Regions
1	63 yrs	M	normal	Cortex
2	73 yrs	F	normal	Cortex
3	35 yrs	F	normal	Cortex
4	30 yrs	F	normal	Cortex
5	35 yrs	M	normal	Cortex
6	11 yrs	F	normal	Cortex
7	3 mon	F	Acute hypoxic ischemic Infarction,	Cortex
8	6 mon	M	old germinal matrix hemorrhage	Cortex
9	14 yrs	M	vascular malformation with hemorrhage, diffuse ischemia	Cortex
10	20 days	F	Hypoxic ischemic injury	Cortex
11	3 mon	M	Hypoxic ischemic injury	Cortex

Supplementary Figure 2. Expression of AIF3 in neurons in vitro and mouse cortex under different pathological conditions. a, TTC staining of C57BL/6 mouse brain at 24 h reperfusion after 2 h MCAO. **b**, Expression of AIF3 in cortical neurons 24 h after NMDA (500 μM for 5 min or 100 μM for 2 h) or Staurosporine (STS, 1 μM) treatment. CSS, control saline solution. PC (positive control), cortical lysate of AIF3 splicing mice. **c**, Expression of AIF3 in cortical neurons at 24 h and 48 h after exposure to OGD for 90 min. PC, cortical lysate of AIF3 splicing mice. **d**, Expression of AIF3 in cortical neurons exposed to 24 h, 48 h and 72 h hypoxia (1% O_2). PC, cortical lysate of AIF3 splicing mice. **e**, De-identified human stroke patients' information.

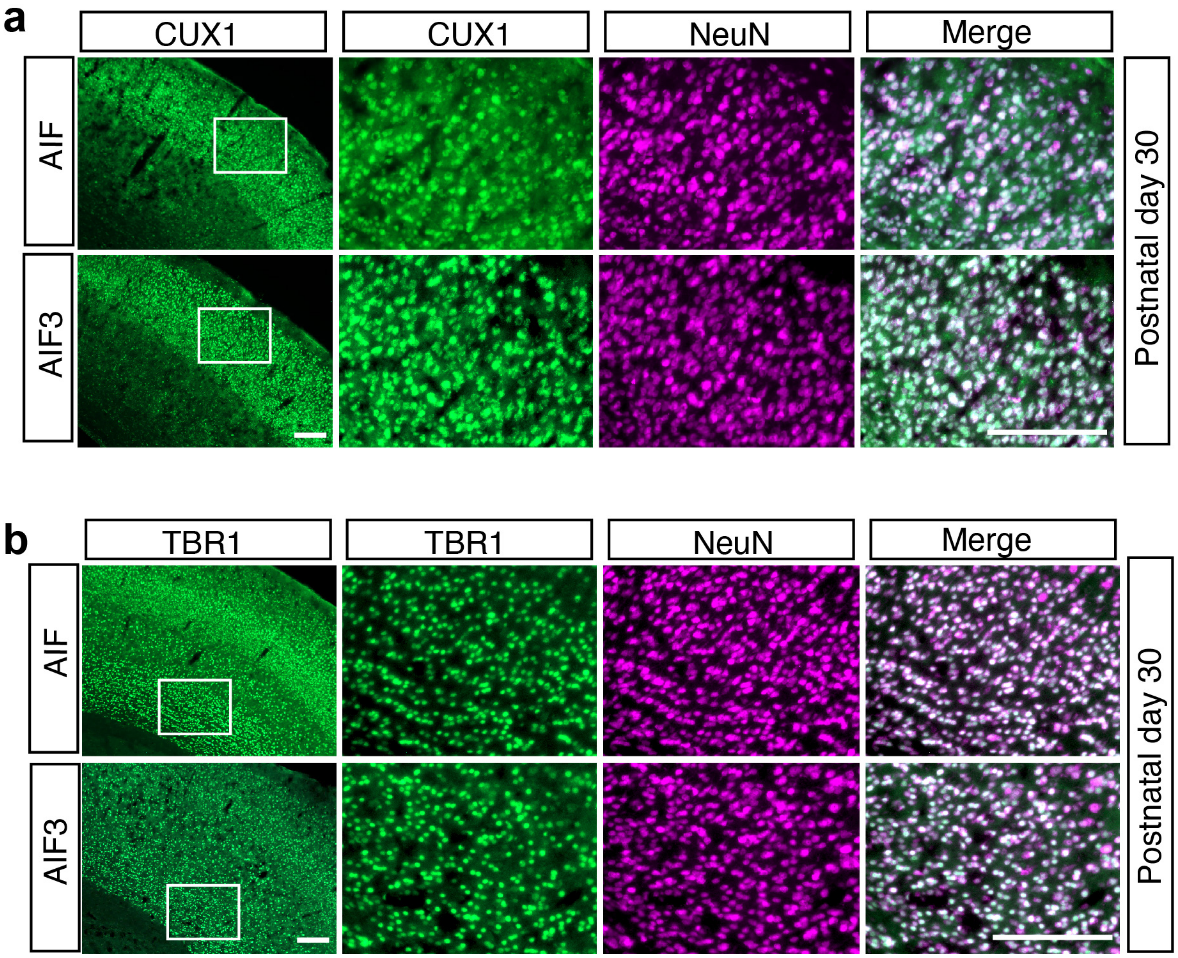

Supplementary Figure 3. Characterization of neuron marker expression in the cortex of AIF3 splicing mice at P30. a, Representative images of CUX1+/NeuN+ neurons in AIF3 splicing mice (AIF$^{fl/Y}$/CamKIIα-iCre+) and littermate control AIF mice (AIF$^{fl/Y}$/CamKIIα-iCre-) at P30. **b**, Representative images of TBR1+ /NeuN+ neurons in AIF3 splicing mice (AIF$^{fl/Y}$/CamKIIα-iCre+) and littermate control AIF mice (AIF$^{fl/Y}$/CamKIIα-iCre-) at P30. Scale bar, 200 μm.
